# Identification of Novel Raft Marker Protein, FlotP in *Bacillus anthracis*

**DOI:** 10.3389/fmicb.2016.00169

**Published:** 2016-02-17

**Authors:** Vikas K. Somani, Somya Aggarwal, Damini Singh, Tulika Prasad, Rakesh Bhatnagar

**Affiliations:** ^1^Laboratory of Molecular Biology and Genetic Engineering, School of Biotechnology, Jawaharlal Nehru UniversityNew Delhi, India; ^2^AIRF, Jawaharlal Nehru UniversityNew Delhi, India

**Keywords:** pathogen, *Bacillus anthracis*, flotillin, SPFH, DRM, FlotP, microdomain, lipid raft

## Abstract

Lipid rafts are dynamic, nanoscale assemblies of specific proteins and lipids, distributed heterogeneously on eukaryotic membrane. Flotillin-1, a conserved eukaryotic raft marker protein (RMP) harbor SPFH (Stomatin, Prohibitin, Flotillin, and HflK/C) and oligomerization domains to regulate various cellular processes through its interactions with other signaling or transport proteins. Rafts were thought to be absent in prokaryotes hitherto, but recent report of its presence and significance in physiology of *Bacillus subtilis* prompted us to investigate the same in pathogenic bacteria (PB) also. In prokaryotes, proteins of SPFH2a subfamily show highest identity to SPFH domain of Flotillin-1. Moreover, bacterial genome organization revealed that Flotillin homolog harboring SPFH2a domain exists in an operon with an upstream gene containing NFeD domain. Here, presence of RMP in PB was initially investigated *in silico* by analyzing the presence of SPFH2a, oligomerization domains in the concerned gene and NfeD domain in the adjacent upstream gene. After investigating 300 PB, four were found to harbor RMP. Among them, domains of Bas0525 (FlotP) of *Bacillus anthracis* (*BA*) showed highest identity with characteristic domains of RMP. Considering the global threat of *BA* as the bioterror agent, it was selected as a model for further *in vitro* characterization of rafts in PB. *In silico* and *in vitro* analysis showed significant similarity of FlotP with numerous attributes of Flotillin-1. Its punctate distribution on membrane with exclusive localization in detergent resistant membrane fraction; strongly favors presence of raft with RMP FlotP in BA. Furthermore, significant effect of Zaragozic acid (ZA), a raft associated lipid biosynthesis inhibitor, on several patho-physiological attributes of BA such as growth, morphology, membrane rigidity etc., were also observed. Specifically, a considerable decrease in membrane rigidity, strongly recommended presence of an unknown raft associated lipid molecule on membrane of BA. In addition, treatment with ZA decreased secretion of anthrax toxins and FlotP expression, suggesting potential role of raft in pathogenesis and physiology of BA. Thus, the present study not only suggest the existence and role of raft like entity in pathophysiology of BA but also its possible use for the development of novel drugs or vaccines against anthrax.

## Introduction

Eukaryotic plasma membrane harbor small (size ~10–100 nm) but complex dynamic assemblies of lipids and proteins constituting membrane microdomains (commonly referred to as lipid rafts), which coordinate membrane signaling and trafficking with raft associated proteins (Bickel et al., 1997; Pike, 2006; Lingwood and Simons, 2010). Moreover, reports on association of lipid raft impairment with various human diseases like Alzheimer, Prion, Parkinson etc., reflect its profound significance in cellular organization as well as physiology (Donovan and Bramkamp, 2009).

Microdomains create heterogeneity on eukaryotic membrane due to high density of specific lipids and proteins, whose stoichiometry in these sub regions make them insoluble in the non-ionic detergents yielding detergent resistant membrane (DRM) and detergent sensitive membrane (DSM) regions (Bickel et al., 1997; Dermine et al., 2001). Flotillin-1, associated with DRMs, has been considered as the bona-fide marker for lipid rafts (Bickel et al., 1997; Dermine et al., 2001; Lingwood and Simons, 2010). Flotillins are conserved, integral membrane proteins with unique SPFH domain (named after Stomatins, Prohibitin, Flotillin, and HflK/C) on its N-terminal followed by Flotillin/oligomerization domain. Flotillin domain has coiled-coil regions which favor its self oligomerization and interaction with other colocalized proteins (Bach and Bramkamp, 2013). It is presumed that interaction between these two domains favors heterogeneous distribution of Flotillins on membrane (Bach and Bramkamp, 2013). In addition, Flotillins have also been shown to be involved in scaffolding of DRMs and recruitment of proteins to lipid rafts to facilitate proper interactions, oligomerization, and functioning such as signal transduction, coupling of membrane-cytoskeleton, and endocytosis (Bickel et al., 1997; Dermine et al., 2001; Krogh et al., 2001; Zhang et al., 2005; López and Kolter, 2010; Zhao et al., 2011; Bach and Bramkamp, 2013). These vital roles of microdomains in membrane organization strongly recommend its need in physiology as well as metabolism.

Till recently, prokaryotes were considered too simple to harbor such complex membrane organization (Bramkamp and Lopez, 2015). However, lipid rafts with homolog of eukaryotic RMP Flotillin-1 have been reported for vital cellular organization and functions such as maintenance of membrane integrity and signaling in non-pathogenic spore forming bacteria, *Bacillus halodurans*, and *B. subtilis* (Zhang et al., 2005; Meile et al., 2006; Donovan and Bramkamp, 2009; López and Kolter, 2010). The role of these bacterial rafts in membrane heterogeneity, sporulation, biofilm formation etc., (Donovan and Bramkamp, 2009; Bramkamp and Lopez, 2015) raises the idea that these might be the niche for numerous proteins involved in crucial bacterial physiological processes. Moreover, in bacteria, lipid raft disruption interferes with vital cellular processes without affecting cell viability and thus, is less likely to exhibit spontaneous mutation-acquired resistance against anti-raft compounds like statins that make these drugs less antimicrobial resistant (Donovan and Bramkamp, 2009; Bramkamp and Lopez, 2015). Commercially available anti-raft compounds have already been proved to be non-toxic to humans even at higher concentrations (Bramkamp and Lopez, 2015). In addition, rafts are also known to harbor sterol like molecules. Therefore, inhibitors of enzymes involved in rafts associated lipid biosynthesis have also been shown to affect microdomain organization and thus, physiology of the organism (López and Kolter, 2010). Hence, investigating existence of rafts in pathogenic microbes is likely to reveal hidden insights into bacterial pathophysiology to develop novel drugs or anti-raft compounds that may possess broad spectrum activity, less antimicrobial resistance and minimal host side effects.

Although, variations might exist in microdomains associated proteins or lipids, but features of Flotillin-like proteins remain conserved throughout. Thus, characterization of Flotillin appears to be the most reasonable approach to explore microdomains in bacteria. In this study, we performed *in silico* analysis to find protein(s) in bacterial pathogens with various primary and secondary structural attributes of Flotillin. The high identity of BAS0525 (FlotP) of *Bacillus anthracis* to above attributes of Flotillin-1 and importance of anthrax in public health globally prompted us to characterize FlotP (Simons and Sampaio, 2011). Also, it has been seen that biosynthesis of bacterial raft associated lipids involve squalene synthase (López and Kolter, 2010) whose known inhibitor Zaragozic acid (ZA) was found to impede the existence and formation of bacterial raft. Thus, to claim existence of raft, a parallel investigation of raft associated lipid molecule in bacteria would be highly recommended. Therefore, in order to further explore raft, effect of this inhibitor on several pathophysiological attributes of *B. anthracis* were also studied.

## Materials and methods

### *In silico* identification of flotillin homolog and its related gene in pathogens

For the identification and selection of the most significant Flotillin homolog harboring pathogens from around 300 different pathogenic bacterial strains, following steps were followed. All sequences were retrieved from KEGG database. Initially, orthologs of SPFH2a domain containing homolog of prokaryotic Flotillin were searched by Clustal W using YuaG of *B. subtilis* as a subject. Since sequences of many of the orthologs showed similarity to *B. subtilis* YuaG, a criterion of presence of SPFH2a domain was taken. The alignment was used as input for the generation of phylogenetic tree between different species by Clustal Ω. CLUSTAL 2.1 was also employed to determine presence of various sub-domains like oligomerization/flotillin and SPFH2a in selected sequences. Oligomerization/flotillin domain identity was taken as second criteria for screening of suitable pathogens selected by initial analysis. Resulting list of bacteria fulfilling the initial two criteria were compared for the presence of NFeD domain harboring gene adjacent to Flotillin homolog. The values for identity matrix at various stages of analysis were calculated by alignment through Clustal Ω. UPGMA method was employed for phylogenetic tree construction. Bootstrapping of phylogenetic tree was performed by MEGA software 6.06 version with 1000 No. of Bootstrap Replications using Poisson model.

### Operon analysis of *flotP* and *nfeD* by RT-PCR

*In silico* prediction of operon was done using DOOR database (Mao et al., 2009). For validating co-transcription of *flotP* and *nfeD*, RNA was first isolated from overnight grown *B. anthracis* cells by trizol method. DNase treatment was given to remove genomic DNA contamination. cDNA was prepared using 2 μg of DNase treated RNA by High fidelity Reverse Transcriptase kit (Applied Biosystem). Co-transcription was then confirmed by PCR using intergenic primers (Fp: TACTCACTGTGATTTATATC, Rp: TAAAATGAGTAGAATTAAAA) covering a sequence of *flotP* and *nfeD.*

### Bacterial strain, media, and culture conditions

Sterne 34F_2_ (pXO1^+^ pXO2^−^) strain of *B. anthracis* was used for all experiments. *Escherichia coli* DH5α and BL21(λDE3) were used for cloning and expression of *flotP*, respectively. For all experiments, *E. coli* and *B. anthracis* Sterne strain were grown in Luria Bertini (LB) and Brain Heart Infusion (BHI) medium containing 1% NaHCO_3_, respectively. Kanamycin (50 μg/ml) and ampicillin (100 μg/ml) were added wherever required. Eighty micrometers of Zaragozic acid (ZA; procured from Sigma) in 95% ethanol was used, for various comparative studies.

### Cloning, expression, purification of FlotP, and production of antibody

Standard procedures were used for cloning. The open reading frames corresponding to BAS 0525 (*flotP*) was identified and retrieved from NCBI. Full length *flotP* (1581 bp) from genomic DNA of *B. anthracis* Sterne was amplified employing forward and reverse primer containing Nco1 and Xho1 restriction sites Amplicon *flotP* was ligated in pET-28a (Novagen) expression vector digested with same respective enzymes to obtain pET-flot with 6x Histidine tag at N- terminal. *E. coli* BL21(λDE3) cells were used for protein expression which was achieved by inducing the culture at O.D_.600*nm*_ ~ 0.6 with the addition of 1 mM IPTG for 6 h at 37°C. Purification of FlotP was done from cytosolic fraction of recombinant *E. coli* BL21(λDE3) under native condition using Ni^2+^—NTA affinity chromatography as described previously (Agarwal et al., 2008). The purified protein was then dialyzed against 10 mM Tris (pH 8.0) and 10% glycerol. Protein concentration was determined by using Bradford reagent (Kruger, 2009) taking BSA as a standard. Purified, dialyzed protein was used to immunize Swiss albino mice of 4–6 weeks, procured from the animal house facility of the university, for raising polyclonal sera against specified protein (Sinha and Bhatnagar, 2010). The antibody titers were calculated using standard endpoint ELISA (Frey et al., 1998). Animal experiments were approved by Institutional Animal Ethics Committee of Jawaharlal Nehru University and were done under standard laboratory conditions at animal facility of the university.

### Protein identification by MALDI

Purified rFlotP was run on SDS-PAG followed by coomassie staining. The protein band corresponding to FlotP was excised manually and the sample is processed as described previously (Savary and Vasu, 2012). The MALDI-TOF analysis was executed by using Bruker Daltonic Ultraflex TOF/TOF system. For peptide search, MASCOT search engine and NCBI non-reduntant database were used.

### Secondary structure prediction and validation

Secondary structure was predicted by CFSSP server. This helped us to define patterns of various sub-regions of protein. Coiled-coil server was employed to determine the most prominent region of interaction or coiled-coil formation with window size of 14, 21, and 28 amino acids. TopPred software was used to predict transmembrane helix and its orientation on the membrane by calculating hydrophobicity index of the entire sequence. Secondary structure of FlotP was again validated by Circular Dichroism Spectroscopy (CDS) which was done by taking rFlotP at a concentration of 0.2 mg/ml in 10 mM phosphate buffer (pH 7.4). Spectra was obtained by using Jasco Corp., J-710 Spectropolarimeter at 25°C using a 1 mm cell, a wavelength scan from 190 to 240 nm at the rate of 20 nm/min. Minimum 10 scans were taken for each sample and its relevant baseline. The resulting averaged baseline spectrum was then deducted from averaged sample spectrum. The signals thus, obtained were used to calculate the molar ellipticity using the formula: θ_m_ = θ_o_ × 100/lc, where,

θ_m_, molar ellipticity;

θ_o_, observed ellipticity;

l, path length;

c, molar concentration.

Analysis was done as described previously (Greenfield, 2006; Rahi et al., 2011).

### *In vivo* expression study

#### Quantitative real time PCR

To analyze the expression of *flotP* at transcript level, qRT-PCR was done at different growth stages according to the growth curve of *B. anthracis* (early exponential, mid exponential, late exponential, and onset of stationary phase). RNA was isolated by trizol method. High capacity cDNA reverse transcription kit (Applied Biosystems) was used for cDNA synthesis taking DNase I treated RNA as a template. PCR reactions were run in ABI PRISM 7500HT sequence detection system from Applied Biosystems. DNA Gyrase gene was taken as internal control for normalization. Result was analyzed by 2^−ΔΔCT^ method. Data was represented as fold change in gene expression profile after normalizing with DNA Gyrase.

For analyzing effect of ZA on *flotP* expression at transcript level, RNA was isolated from *B. anthracis* culture grown overnight with or without ZA. cDNA preparation and qRT-PCR analysis was done as described above.

#### Immunoblotting

*B. anthracis* cells were allowed to grow till OD reached 0.3, 0.6, 0.9, 1.2, and 1.5 in BHI media. The cells were then pelleted down and subjected to sonication at 30% amplitude for 20 min (750 W Sonic Vibra Cell Sonicator). The lysates were then collected and quantified for their total protein content by Bradford reagent (Sigma). Volume of lysate containing equal protein was then mixed with SDS-loading dye and run on 12% SDS-PAGE followed by immunoblotting using nitrocellulose (NC) membrane. The blot was then developed using protein specific polyclonal antisera as a primary antibody and goat-antimouse IgG-alkaline phosphatase conjugated as secondary antibody.

For analyzing effect of ZA on FlotP expression at protein level, *B. anthracis* was allowed to grow overnight with or without ZA. Cell lysate preparation and immunoblotting was done as described above.

### Localization studies of FlotP

#### *In Silico* tools

PSORTb and TMHMM were used for predicting localization of FlotP in *B. anthracis.* TopPred was used for determining hydrophobicity of the protein.

#### Flow cytometric analysis

FACS analysis was done as described previously (Matta et al., 2010) by using FACS Calibur (Beckton Dickinson, Heidelberg, Germany). Anti-rFlotP at a dilution of 1:200 was used. Pre-immune sera (1:200) and only cells were taken as negative control. Anti-rGAP A was used as positive control with the dilution 1:200. IgG-FITC labeled secondary antibody was used with the 1:100 dilution for all samples. Propidium iodide staining was used to differentiate between live and dead bacterial cells. Ten thousand cells were taken to analyze fluorescence and log-side scatter and log-forward scatter dot plot were used to detect the cells. Cell debris and larger cell aggregates were excluded by gating.

#### Immunolocalization studies

##### Confocal fluorescence microscopy

Heterogeneous distribution of FlotP was analyzed by confocal microscopy using anti-rFlotP antibody. For this, B. anthracis cells were allowed to grow to OD_600*nm*_ ~ 1.2, pelleted down and washed with 1X PBS thrice. Cells were then fixed using 4% paraformaldehyde in 1X PBS for 30 min at room temperature (RT). Washing was then done thrice with 1X PBS. Blocking of cells was done using 2% BSA for 1 h followed by three washes with IX PBS. Cells were incubated with anti-rFlotP antibody at a dilution of 1:200 overnight at 4°C, followed by PBS washing. Cells were then incubated with anti-mouse IgG-FITC labeled antibody (1:100) for 2 h at RT and again followed by three washes with PBS. Finally, the cells were mounted on glass slide and visualized under Olympus FluoView FV1000 Laser Scanning Confocal Microscope.

##### Immunogold electron microscopy

For imunogold localization studies, B. anthracis cells were harvested from stationary phase culture and fixed with 0.8% glutaraldehyde and 4% paraformaldehyde for 2 h at 4°C followed by washing with 1X PBS. Fixed cells were then blocked with 2% BSA and then imunostained with 1:200 dilution of anti-rFlotP followed by three consecutive PBS washes. The cells were then incubated with gold (10 nm) conjugated anti-mouse secondary antibody at a dilution of 1:100. Three washes with PBS were again given to cells which were then mounted on copper coated grids. Immunostained cells were then visualized using a Jeol 2100F transmission electron microscope (Jeol Analytic Instruments) with the acceleration voltage of 120 KV.

#### Submembrane localization in detergent resistant (DRM) and detergent sensitive (DSM) fraction

*B. anthracis* cells were allowed to grow overnight at 37°C in BHI. Cells were collected by centrifugation, washed twice with 1X PBS and finally resuspended in buffer containing 50 mM Tris (pH-8.0) at a cell concentration of nearly 10^10^ cells/ml. Sonication was then done for 30 s, followed by centrifugation at 4°C. The supernatant containing cytosolic fraction was collected. The pellet was then washed with 50 mM Tris (pH-8.0) followed by resuspension in extraction solution (8 M urea, 4% w/v CHAPS, 40 mM Tris, 2% DTT, and 0.2 w/v Bio-Lyte 3/10) and incubated for 30 min at RT. Supernatant containing the membrane fraction was then collected. The membrane fraction was further fractionated into DRM and DSM using CellLytic MEM protein extraction kit (Sigma) as described previously (López and Kolter, 2010). Quantitation of protein was done by Bradford assay. Equal dilutions of protein from each fraction were then loaded on 12% SDS-PAGE, immunoblotted on NC membrane and detected as described above.

### Growth analysis

Effect of ZA on growth of *B. anthracis* was assessed by inoculating 1% inoculum from overnight grown culture into fresh BHI containing 1% NaHCO_3_ with or without ZA. Aliquots were collected from each tube after every hour upto 9 h. Optical density at 600 nm was measured with required dilution periodically.

### Morphology analysis

For microscopic analysis, stationary phase *B. anthracis* cells grown in BHI containing 1% NaHCO_3_ with or without ZA were taken, washed with 1X PBS thrice followed by fixation in 1% (v/v) glutaraldehyde for 2 h at 4°C. Washing was done again with 1X PBS. Washed cells were then used for SEM analysis. For analysis, sample was prepared as described previously (Klee et al., 2006). Visualization was done using **Zeiss EV040** scanning electron microscope (SEM) at Advance Instrumentation Research Facility (AIRF), JNU. Image J software was used for measuring cell dimensions.

### Determination of change in membrane fluidity of *B. anthracis*

*B. anthracis* cells were grown in the presence or absence ZA till OD_600*nm*_ ~ 0.8. Cells were harvested by centrifuging at 10,000 rpm for 10 min followed by washing with 1X PBS twice. The cells were then fixed with 4% paraformaldehyde. Fixed cells were washed again with 1X PBS followed by its dilution in 1X PBS so that the resulting cell count would be equal to 3^*^10^8^. Fluoresence polarization (**Steady state fluorescence)** and Time Resolved Florescence Spectrometry (TRFS) were exploited to study effect of inhibitors of sterol biosynthetic pathway on the physical state of the membrane of *B. anthracis* cells.

#### Fluorescence polarization

Fluorescence polarization which gives a measure of membrane fluidity was assessed using fluorescent probe 1, 6-diphenyl- 1, 3, 5-hexatriene (DPH). The method described earlier (Prasad et al., 2006) was followed with little modifications. PBS washed cells were diluted to 1^*^10^8^ cells/ml and were incubated with 2 μm DPH for 2 h at 30°C. The polarization ratio (p) was then calculated on Perkin-Elmer LS55 spectrofluorimeter (excitation 360 nm, emission 426 nm, and slit size 10 nm for both excitation and emission) as follows (Shinitzky and Barenholz, 1978).

$$\text{p}=\frac{{\text{I}}_{\text{VV}}-({\text{I}}_{\text{VH}}\times \text{G})}{{\text{I}}_{\text{VV}}+({\text{I}}_{\text{VH}}\times \text{G})}$$

where, I_VV_ = Corrected fluorescence intensity obtained with excitation by vertically polarized light and emission detected by analyzer oriented vertically to the direction of polarized excitation light, I_VH_ = Corrected fluorescence intensity obtained with excitation by vertically polarized light and emission detected by the analyzer oriented horizontal to the direction of polarized excitation light.

*Grating Factor* G, the correction for optical components of the instrument is calculated as I_HV_/I_HH_ where subscripts HV and HH indicate the corrected fluorescence intensity values obtained with horizontal-vertical and horizontal-horizontal orientations for the polarizer and analyzer in that order, respectively.

#### TRFS

TRFS was done using FL920 Edinburgh spectrofluorimeter which was equipped with a xenon arc lamp, a polarizing device and a 375-nm laser diode (NanoLED 375 L, Horiba Ltd.) operated with a pulse frequency of 1 MHz. During the experiment photobleaching was avoided by using extremely low power (6–7 μW).

Decays in nanosecond timescales of DPH were measured in time correlated single photon counting (TCSPC) setup (FL920, Edinburgh Instruments, UK). Samples were excited at 375 nm using picosecond diode laser (pulse width ~100 ps). Fluorescence were dispersed in a monochromator and then collected by a MCP-PMT detector. The time-resolution of TCSPC setup of ~100 ps was determined by measuring the Instrument Response Function (IRF) using LUDOX solution. DPH labeled cells were placed in a quartz cuvette. Fluorescence was emitted at 426 nm and the time 100 ns was split into 4096 channels. Decay was measured at magical angle for 5000 peak counts. The G factor was determined by measuring I_HH_ and I_HV_ between the ranges 400–600 nm at five repeats. The anisotropy decay was determined by convolving the I_VH_ and I_VV_.

### Study of effect of ZA on secretion of toxins (PA, LF, and EF)

The level of primary virulence factors like PA, LF, and EF secreted in the presence of different inhibitors were quantitated using ELISA as described previously (Aggarwal et al., 2015). Briefly, the media supernatants from BHI with 1% NaHCO_3_ grown *B. anthracis* cultures containing various inhibitors were first concentrated. Equal volumes of concentrated supernatants were coated in 96-well microtitre plate and kept overnight at 4°C. Plate was then blocked with 2% BSA for 1 h at RT followed by three consecutive washings using 1X PBST containing 0.05% Tween-20. The plate was then incubated with monoclonal goat anti-PA, anti-LF, and anti-EF antibodies in respective wells at a dilution of 1: 15,000, 1: 5000, 1:7000, respectively, and kept at RT for 1 h. The plate was given again three successive washes with 1X PBST followed by incubation with anti-goat-IgG-HRP (1:10,000) dilution at RT for 1 h. Plate was then again washed and colorimetric analysis was done by using TMB substrate and OD was measured at 630 nm.

### Statistical analysis

For analyzing statistical data, Microsoft excel was used. The data was expressed as mean ± S.D. otherwise indicated. The experiments were repeated at least three times and for analyzing the significance (*p*-value) between different data points, unpaired student *t*-test was used. *p*-value < 0.05 was considered as statistically significant.

## Results and discussion

### Flotillin homolog in pathogenic bacteria

**Figure 1:**
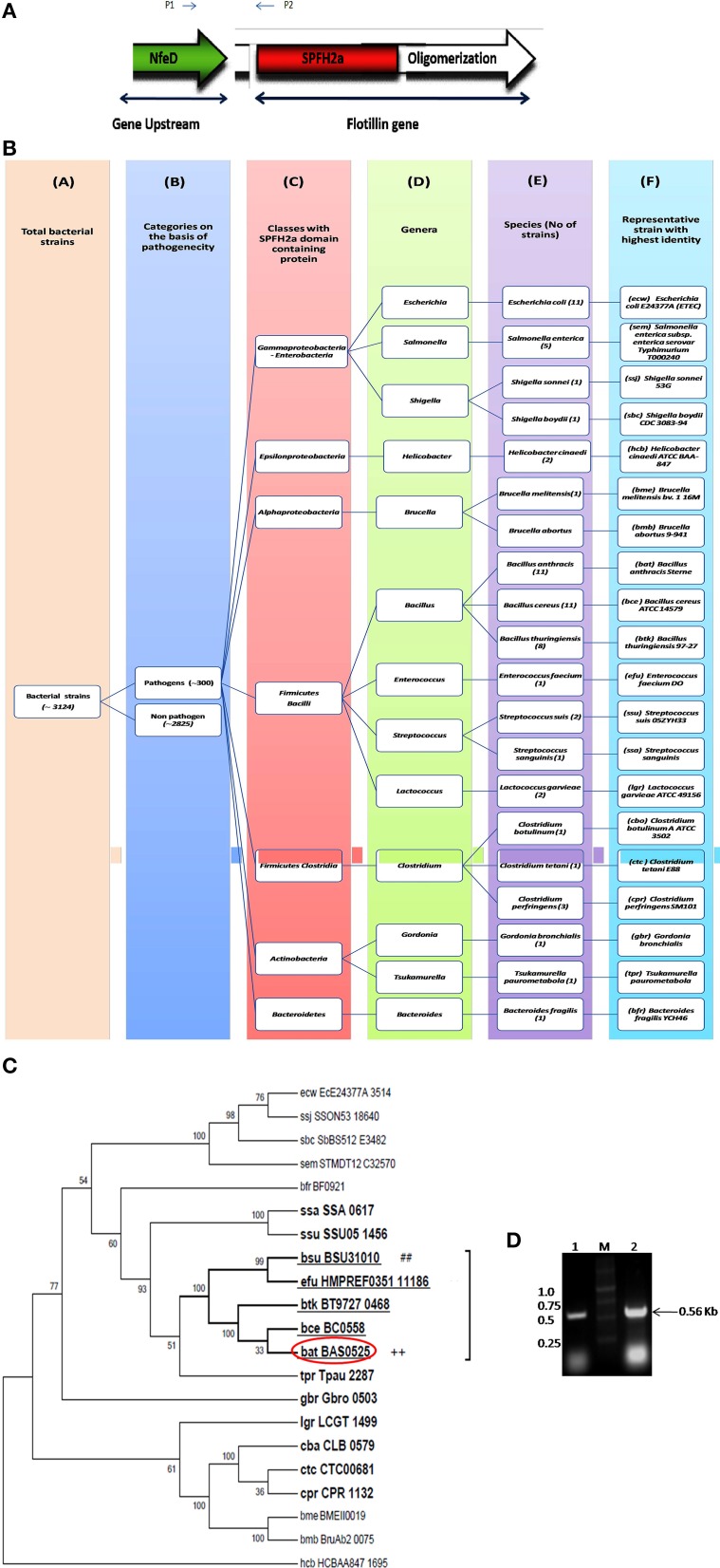
**Flotillin-associated domains and identification of flotillin homologs in pathogens. (A)** Representation of various domains associated with Raft Marker Protein or Flotillin-1. **(B)** Schematic representation of 20 shortlisted bacterial pathogens containing proteins of SPFH2a superfamily, each representing individual species. Columns: C, Classes; D, Genera; E, Species; F, Selected strain showing maximum identity with SPFH2a domain. Number in bracket in column E represents total number of strains in that particular species showing identity with SPFH2a domain. **(C)** Phylogenetic tree showing distance between various PB containing proteins with SPFH2a domain of YuaG of *B. subtilis* (##), characteristic feature of prokaryotic Flotillin. Among all SPFH2a domain harboring pathogens, the species in bold showed presence of oligomerization domain also. Among them, underlined species showed highest identity with Nfed domain. Encircled species with ++ is finally selected for further charecterization. **(D)** Co-transcription of Bas0524 (NfeD domain containing gene) and Bas0525, *flotP.* Amplification with primers P1 and P2 [as represented in **(A)**] was analyzed when cDNA (Lane 1) and genomic DNA (Lane 2) were taken as template. M represents Marker.

Flotillin-1 is a member of SPFH superfamily harboring evolutionary conserved SPFH (Stomatin, Prohibitin, Flotillin, and HflK/C protein) domain which shows affinity for membrane microdomains (Tavernarakis et al., 1999; Zhang et al., 2005; Browman et al., 2007). Prokaryotic SPFH super family has been subdivided into 12 subfamilies based on the sequence and operon structure (Hinderhofer et al., 2009) out of which SPFH2a subfamily of proteins show highest similarity to Flotillin-1. A recent study reported presence of Flotillin homolog, YuaG of SPFH2a subfamily as raft marker in a non-pathogenic bacteria *B. subtilis* (López and Kolter, 2010; Dempwolff et al., 2012; Mielich-Süss et al., 2013). Considering the fact that membrane microdomains harbor variety of signaling proteins, it is of utmost interest to investigate presence of such SPFH2a domain harboring proteins in PB as a key step toward investigation of membrane microdomains in infectious bacteria. This study may unveil some unknown aspects of signaling mechanisms as well as pathophysiology of the pathogens. For identification of the best homolog of raft marker-Flotillin-1, 3 different domains namely SPFH2a, and Flotillin/oligomerization domains of the candidate gene and Nfed domain of upstream gene (belongs to same operon) were taken into consideration. A pictorial representation of these domains taken for the identification of Flotillin-like gene in bacterial pathogens are given in Figure 1A.

Homology search for SPFH2a domain of YuaG of *B. subtilis* has been performed in 300 PB strains showed 66 strains containing proteins with SPFH2a domain that belonged to 20 bacterial species. Since, many of these 66 strains belonged to the same species; therefore, the strain from the particular speciesshowing highest similarity to SPFH2a domain was selected as the representative for that species. On this basis, we took a representative pathogen from each of 20 different species for further analysis (Figure 1B). The similarity and distance between all bacterial pathogens harboring SPFH2a domain containing proteins have been represented by the phylogenetic tree in Figure S1. Figures S2A,B, respectively, list 66 bacterial pathogens along with their respective KEGG codes, length of proteins and SPFH2a domain alignment of all pathogens.

Since, Flotillin-1 possesses a characteristic oligomerization/Flotillin domain, our next step was to find this domain in the proteins of shortlisted 20 pathogens. Phylogenetic analysis (Figure 1C) and percent identity matrix (Table 1) further identified only 12 pathogens with more than 25% identity with this domain and interestingly they were all gram positive. Among these, eight were firmicutes from genus *Bacillus* and showed more than 40% identity.

**Table 1:** **List of pathogens showing identity with Flotillin/Oligomerization domain of hsa_10211, Flotillin-1 of *Homo sapiens* (taken as reference)**.

**S.no**.	**Species**	**Percent Identity(%)**
1.	*Lactococcus garvieae* ATCC 49156 (lgr_LCGT_1499)	38.46
2.	*Clostridium perfringens* SM101 (cpr_CPR_1132)	33.33
3.	*Clostridium tetani* E88 (ctc_CTC00681)	31.25
4.	*Clostridium botulinum* A ATCC 19397 (cba_CLB_0579)	34.78
5.	*Gordonia bronchialis* (gbr_Gbro_0503)	32.11
6.	*Tsukamurella paurometabola* (tpr_Tpau_2287)	30.22
7.	*Streptococcus sanguinis* (ssa_SSA_0617)	29.86
8.	*Streptococcus suis* 05ZYH33 (ssu_SSU05_1456)	29.17
9.	*Bacillus thuringiensis* 97-27 (btk_BT9727_0468)	42.36
10.	*Bacillus cereus* ATCC 14579 (bce_BC0558)	42.36
11.	*Bacillus anthracis* Ames (ban_BA_0557)	42.36
12.	*Enterococcus faecium* DO (efu_HMPREF0351_11186)	40.28

In prokaryotes, Flotillin homologs have been shown to be a part of the operon which also contains upstream of Flotillin, a gene with NfeD domain known to help in anchoring of Flotillin within lipid rafts (López and Kolter, 2010; Dempwolff et al., 2012). In non-pathogenic *B. subtilis*, colocalization of YuaF (NFeD2), and YuaG and their influence on mutual localization appears to favor interplay between Flotillin and NFeD within raft assemblies (Krogh et al., 2001). Therefore, identification of pathogenic bacteria containing Flotillin homolog and NfeD gene in the same operon merits attention. Upon examining the percent identity of NFeD domain in the upstream region of the candidate genes selected for above 12 gram positive bacteria taking *B. subtilis* YuaF as reference, only four were found to show more than 30% identity (Figure 1C, Table 2). Among the screened organisms, maximum identity (37.36%) was observed for both *B. anthracis* and *B. thuringiensis*. Of special mention is the fact that *Enterococcus faecium* displayed significant identity value of 35.29% even without containing the desired domain (Figure S3). Among the above two pathogens, *B. anthracis*, a potential biological warfare agent, appears to be an ideal pathogen for such study in order to address global health concerns. Therefore, Flotillin homolog in *B. anthracis*, Bas0525 (hereafter referred to as FlotP) was further investigated to study rafts in pathogenic bacteria.

**Table 2:** **List of pathogens showing significant identity with NFeD domain harboring gene, bsu_BSU31020 of *B. subtilis***.

**S.no**.	**Species**	**Percent Identity (%)**
1.	^*^Enterococcus faecium DO (efu_HMPREF0351_11185)	35.29
2.	*Bacillus cereus ATCC* 14579 (bce_BC0557)	36.78
3.	*Bacillus thuringiensis* 97-27 (btk_BT9727_0467)	37.36
4.	*Bacillus anthracis* Sterne (bat_BAS0524)	37.36

* *Enterococcus faecium showed identity but lacked desired NFeD domain (Figure S3)*.

### Bas 0525 (*flotP*) and Bas0524 (*nfeD* harboring gene) co-transcribes in the form of operon

Studies done so far, our *in silico* predictions as described above as well as DOOR database for operon analysis suggested the co-transcription of Bas0525 *(flotP)* and Bas0524 (*nfeD* harboring gene) in the form of operon at genomic level. Thus, to validate the genomic organization of Bas0525-0524, operon analysis was done by RT-PCR using intergenic primers, represented by P1 and P2 in Figure 1A. On analysis, a band corresponding to the size (~560 bp) of the amplified product was observed with both genomic DNA as well as cDNA (Figure 1D) confirming the genomic organization of the two genes in operon. The result is in consistence with what observed in *B. subtilis* where co-transcription of Flotillin homolog and NfeD harboring gene was found to have a potential role in proper localization of Flotillin homolog on the bacterial membrane (López and Kolter, 2010; Dempwolff et al., 2012).

### MALDI confirmed Bas0525 encoded protein as flotillin homolog

**Figure 2:**
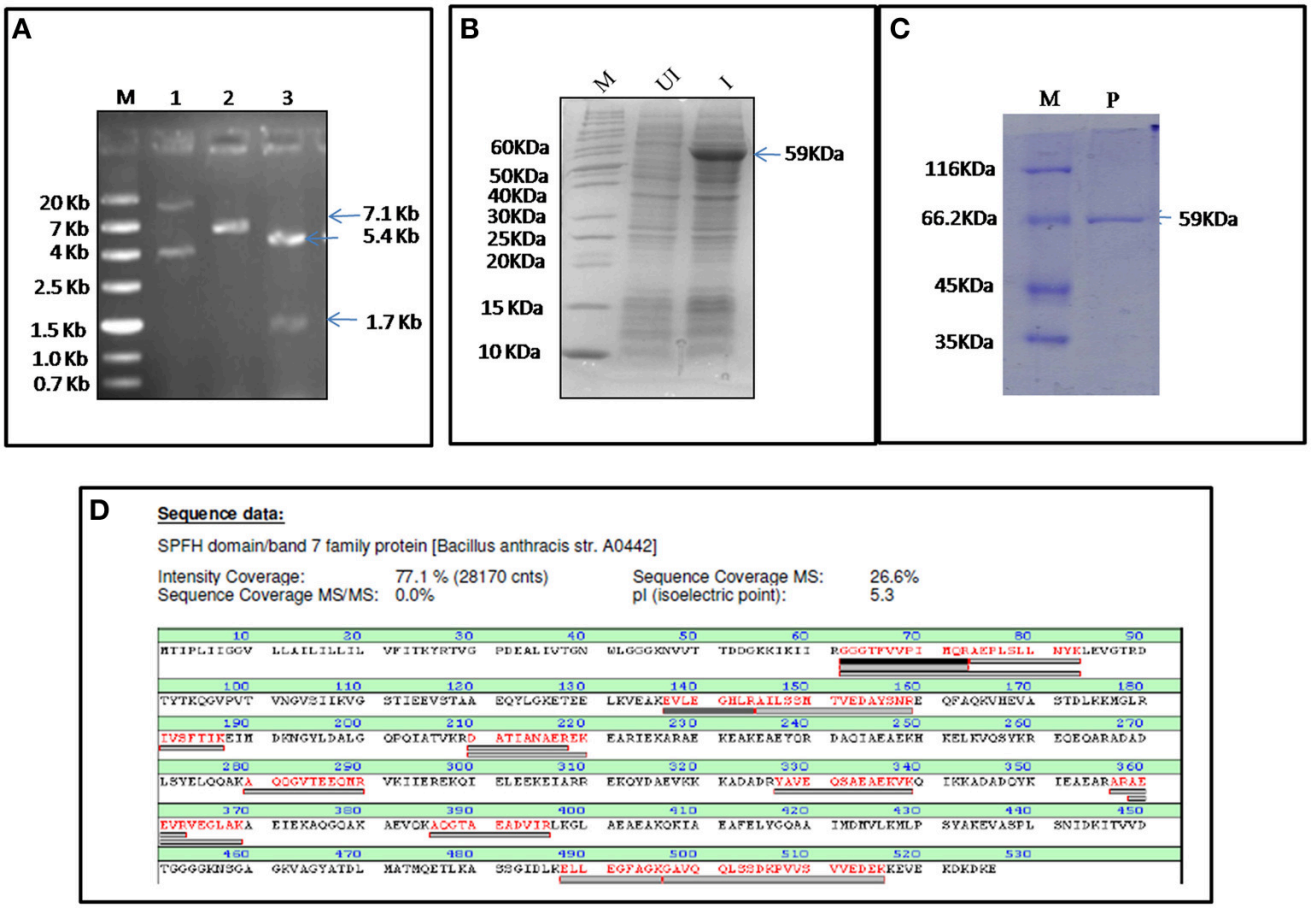
**Cloning, expression, and purification of FlotP. (A)** Clone confirmation by restriction digestion of pET28a-*flotP*. Double digestion (DD) confirmed the presence of insert by fall out at 1.7 Kb corresponding to *flotp* size. Lanes: M- Marker (ZipRuler™; #SM1373), 1- pET28a-*flotP*, 2- Single digest, 3-double digest. **(B)** Overexpression of FlotP in BL21 (λDE3) *E. coli* cells. Lanes: M, Marker (Pageruler unstained; #26614); UI, Uninduced, and I, Induced. **(C)** Purification by Ni-NTA chromatography. Lanes: M, Marker (Pierce™ Unstained Protein MW Marker; #26610); P, Purified Protein. **(D)** MALDI analysis showing the sequence of the protein with the peptide coverage marked in red. The peptide hits showed highest similarity to SPFH domain BAND 7 family protein of *B. anthracis* i.e., FlotP.

FlotP was amplified from *B. anthracis* genome by gene specific primers yielding a 1581 bp amplicon which was further cloned in pET-28a(+) expression vector adding extra nucleotides corresponding to hexa histidine tag (Figure 2A). The rFlotP was allowed to express in BL21(λDE3) cells in the presence of 1 mM IPTG (Figure 2B). Expressed protein corresponding to ~60 KDa was purified from cytoplasmic fraction by Ni-NTA chromatography (Figure 2C). rFlotP was further confirmed by MALDI analysis (Figure 2D).

### *flotP* of *B. anthracis* contains predominantly α-helical and coiled-coil structure

**Figure 3:**
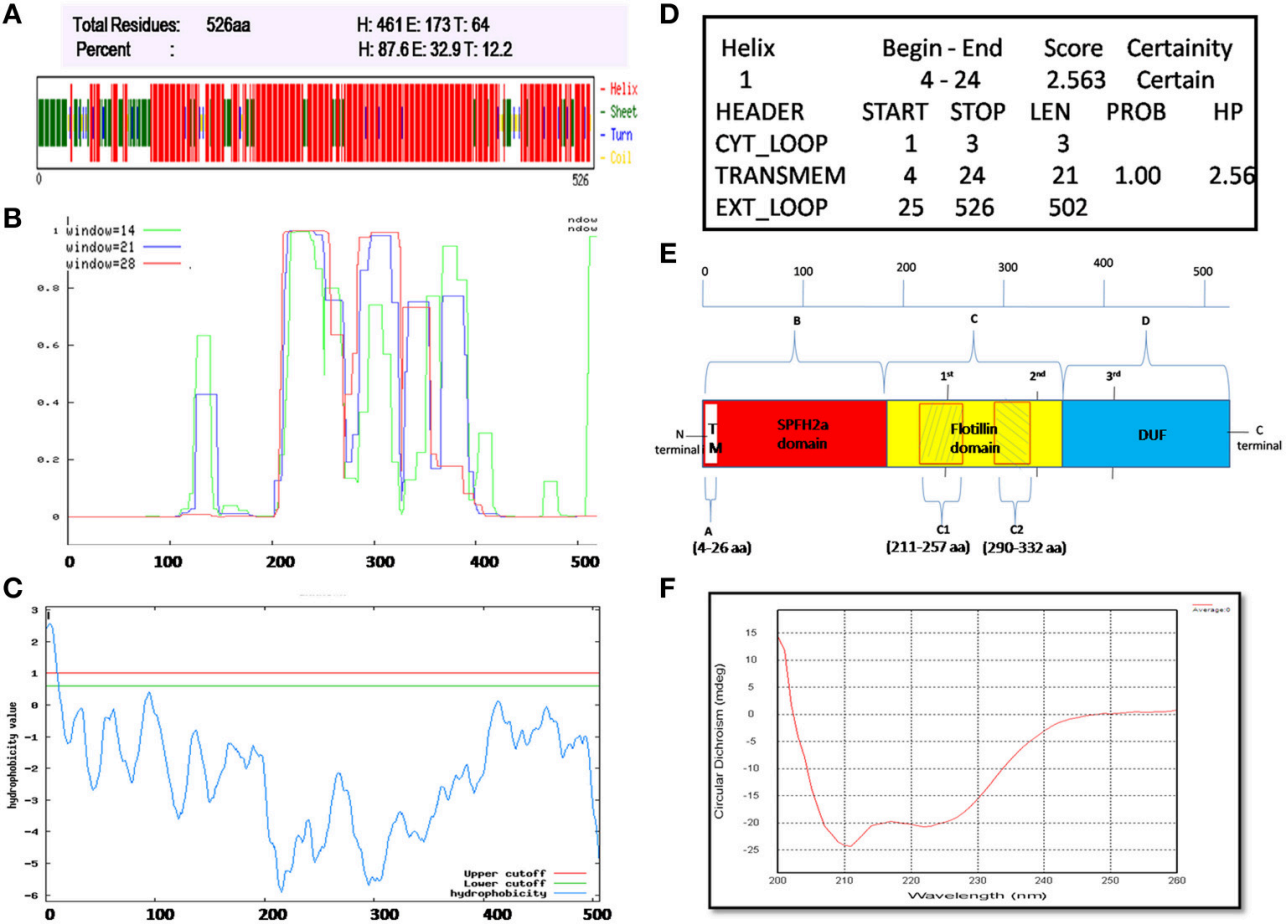
**Prediction and validation of FlotP Topology and Secondary structure. (A)** Secondary structure by CFSSP server showing high percentage of α-helical structure (H-Helix; E-Sheet; T-Turns). **(B)** Prediction of coiled-coil region by Coils server with each size of aa windows. **(C)** Analysis of hydrophobicity index value by TopPred server predicting the presence of transmembrane region. Y-axis of the graph represents hydrophobicity value predicting the possible location of the particular amino acid stretch of the protein. Value > 1 indicate cytoplasmic fragment; 0–1, transmembrane; < 0, membrane. **(D)** Prediction of various sub regions by TopPred with their sub-cellular locations. **(E)** Schematic representation depicting secondary structure and topology of FlotP. Region A in white color represents transmembrane (TM) region (aa 4–26), region B in red color represents SPFH2a domain- characteristic of prokaryotic Flotillin (aa 34–203), region C in yellow color indicates Flotillin or oligomerization domain (aa 203–365) and region D in blue shows domain with unknown function (DUF; aa 365–495). The two shaded boxes (C1- aa 211–257 and C2- aa 290–332) in yellow region C shows two high probability regions of coiled coil or trimer/dimer or interaction. Characteristic 1st, 2nd, and 3rd AEAE repeats are indicated at positions aa 335, 245, and 405. **(F)** CD spectra of purified rFlotP protein showing absolute probability of α-helix secondary structure.

Flotillin-1 consists of α-helical region predominantly (Song et al., 2011). To determine secondary structure of *B. anthracis* FlotP, both *in silico* and *in vitro* analysis was performed in this study. CFSSP Server predicted presence of 66.73% α-helix region in the protein (Figure 3A). However, 95% of Flotillin/oligomerization domain comprised of α-helices favoring coiled-coil formation. The presence of coiled-coil regions was confirmed by COILS Server (with window size 14, 21, and 28) which suggest the presence of two coiled-coil regions between amino acid (aa) 211–257 and 290–332 (Figure 3B). Previous studies report that coiled-coil domains are essential for proper localization, oligomerization, and protein-protein interactions in rafts to facilitate functioning of Flotillin-like proteins in bacteria (Krogh et al., 2001; Zhang et al., 2005). Diverse cellular processes associated with Flotillin assemblies, instead of being lipid dependent have been found to depend on the interactions mediated by coiled-coil domain with other raft associated proteins (Krogh et al., 2001). Moreover, cumulative analysis predicted topology of FlotP similar to mitochondrial prohibitin (Sanz et al., 2003) with transmembrane helix (Figures 3C,D) i.e., N-terminal region toward cytoplasm and C-terminal exposed outwards (Figure 3E). The transmembrane region or hydrophobic stretch between aa 4–26 is likely to facilitate the membrane association. C-terminus of FlotP contains a conserved characteristic Flotillin domain with ala-glu-ala-glu (AEAE) repeats (Figure 3E) favoring the formation of coiled-coil like structures similar to eukaryotic Flotillin (Kurrle et al., 2012). CDS of purified protein confirmed α-helical secondary structure of FlotP (Figure 3F).

### *B. anthracis* flotP is membrane localized

Lipid raft and associated proteins have been reported to be localized on cell membrane (Lingwood and Simons, 2010). Localization of *B. anthracis* FlotP was determined through both *in silico* and *in vitro* analysis. PSORTb (Gardy et al., 2005) and TMHMM (Krogh et al., 2001) softwares predicted membrane localization for FlotP (Figures S4A,B). In addition, hydropathy index determination by TopPred (Krogh et al., 2001) was in agreement with YuaG (Flotillin homolog) of *B. subtilis*, confirming the presence of hydrophobic or transmembrane region (Figures 3C,D)

**Figure 4:**
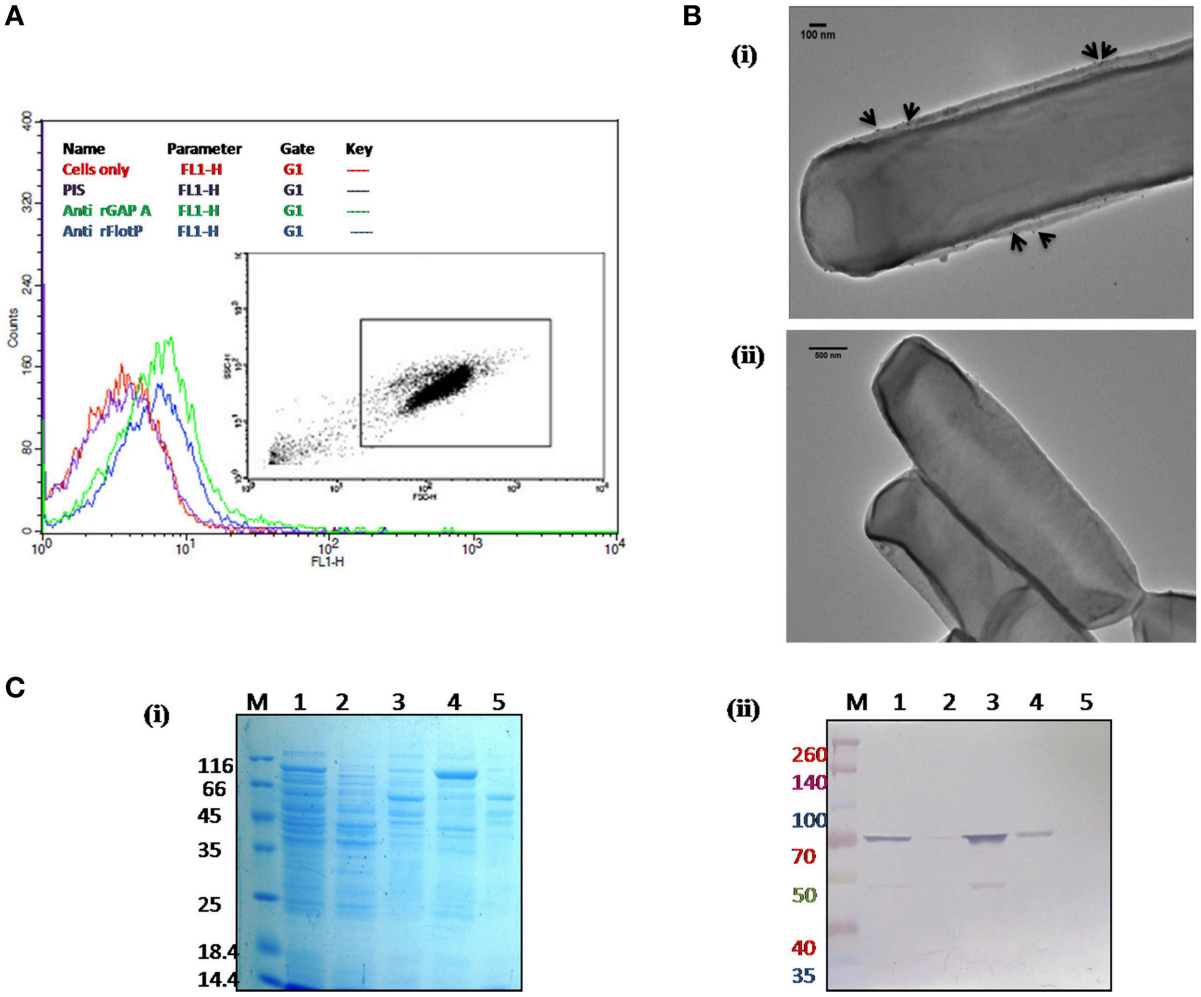
**Localization of *B. anthracis FlotP.* (A)** Flow cytometric analysis for *B. anthracis* cells treated with 1:200 dilution of each anti-rFlotP IgG. anti-rGapA, Pre-immune serum, and only cells were taken as control. After staining with FITC-labeled secondary antibody (1:100), fluorescence was analyzed in FL1 and displayed as a histogram. Inset shows the gated population inside a dot plot between log of both forward scatter (size) and side scatter (granularity/shape). **(B)** Immunogold localization of FlotP on the membrane of *B. anthracis.* Whole vegetative cell of *B. anthracis* was used for TEM analysis. Panel **(i)** The black arrows indicated the gold particles around the membrane of the cell when anti-rFlotP was used as primary antibody. Images were captured at magnification of 12,000x **(ii)** No dots were observed when pre-immune sera was used as primary antibody. Images were captured at magnification of 6000x **(C)** Localization by immunoblotting. Equal amounts of proteins (3 μg) from each fraction were loaded in respective lanes. Panel **(i)** indicates Coommasie stained SDS-PAGE with all fractions and panel **(ii)** shows immunoblotting with anti-rFlotP. Lanes: 1, cellular lysate; 2, cytosolic fraction; 3, membrane fraction after ultracentrifugation; 4, DRM; 5, DSM; and M, Marker.

In order to determine the subcellular localization of FlotP *in vivo*, flow cytometric analysis was done using rFlotP antiserum raised in mice. The data showed positive shift in anti-rFlotP treated bacterial cells in FL1 quadrant which was absent in bacterial cells only (without treating with pre-immune sera) as well as cells treated with pre-immune sera (Figure 4A). Glyceraldehyde 3 phosphate dehydrogenase (GAPDH) is a well-known surface protein of *B. anthracis* (Matta et al., 2010). Thus, for flow analysis, anti-rGapA (polyclonal sera against one of its isoform) was taken as a positive control which showed a positive shift confirming the surface localization of FlotP (Figure 4A). Figure S5 showed histogram statistics for flow analysis.

In order to visualize the FlotP localization on the membrane of *B. anthracis*, immunogold transmission electron microscopy of stationary phase cells was done. Upon visualization, the cells treated with anti-rFlotP and subsequently with Gold conjugated anti-mouse IgG, were found to have gold particles attached with the cell membrane (Figure 4Bi) which were found to be absent in the cells treated with pre-immune sera and then gold conjugated secondary antibody (Figure 4Bii). This further validated our previous observation.

In addition, immunoblotting analysis of various subcellular fractions of *B. anthracis* was also carried out (Figure 4C) confirming the membrane localization of FlotP.

Presence of Flotillins on the plasma membrane probably allows it to act as an interface between intracellular signaling proteins, signaling receptors, and cytoskeleton to support intra- and inter-cellular communication and activate diverse physiological processes (Donovan and Bramkamp, 2009; Dempwolff et al., 2012).

### Heterogenous as well as punctuate distribution of flotP on the plasma membrane and its association with DRM fraction favors microdomain existence in *B. anthracis*

Insolubility in the non-ionic detergents due to association with DRMs is a characteristic property of lipid rafts (Brown, 2002; Magee and Parmryd, 2003). Raft based membrane heterogeneity is maintained at nanoscale to form more stable/ordered membrane assemblies which allows partitioning into functionalized sub-compartments (Bickel et al., 1997). The dynamic liquid-liquid immiscibility of DRMs serves as the basis for the concept of sub-compartmentalization in rafts to focus and coordinate membrane bioactivity (Simons and Sampaio, 2011).

DRMs having lipid raft associated proteins can be isolated by floatation on sucrose density gradients owing to their low density and high lipid-to-protein ratio (Brown, 2002; López and Kolter, 2010). For this study, cell membrane fractionations were performed based on the solubility in non-ionic detergents to separate proteins associated with DRM fractions. On analyzing the DRMs and DSMs of *B. anthracis* on SDS-PAGE, a significant difference in their respective protein profile was observed which clearly implied heterogeneous distribution of proteins with associated macromolecules on *B. anthracis* membrane (Figure 4Ci). Moreover, FlotP was detected in the DRM fraction using immunoblotting with anti-rFlotP (Figure 4Cii). Figure S6 showed the immunoblotting analysis taking DNA Gyrase and GAPDH as a negative and positive controls, respectively for membrane localization. This result was analogous to the previous reports for Flotillin in eukaryotic lipid rafts, directly favoring our prediction for presence of raft like feature in *B. anthracis*.

Moreover, in prokaryotes, lipid rafts have been found to be involved in supporting the bacteria to perform or coordinate its vital functions. In *B. subtilis* a sporulation histidine kinase KinC which is involved in biofilm signaling, shows enhanced activity in the presence of YuaG (Donovan and Bramkamp, 2009). Proteins required for biofilm formation, attachment, signaling and adhesion are found to be present in DRMs in the bacterial pathogen, *Staphylococcus aureus* (Donovan and Bramkamp, 2009). Similar cellular organization thus might be common for all SPFH2a harboring pathogenic bacteria.

**Figure 5:**
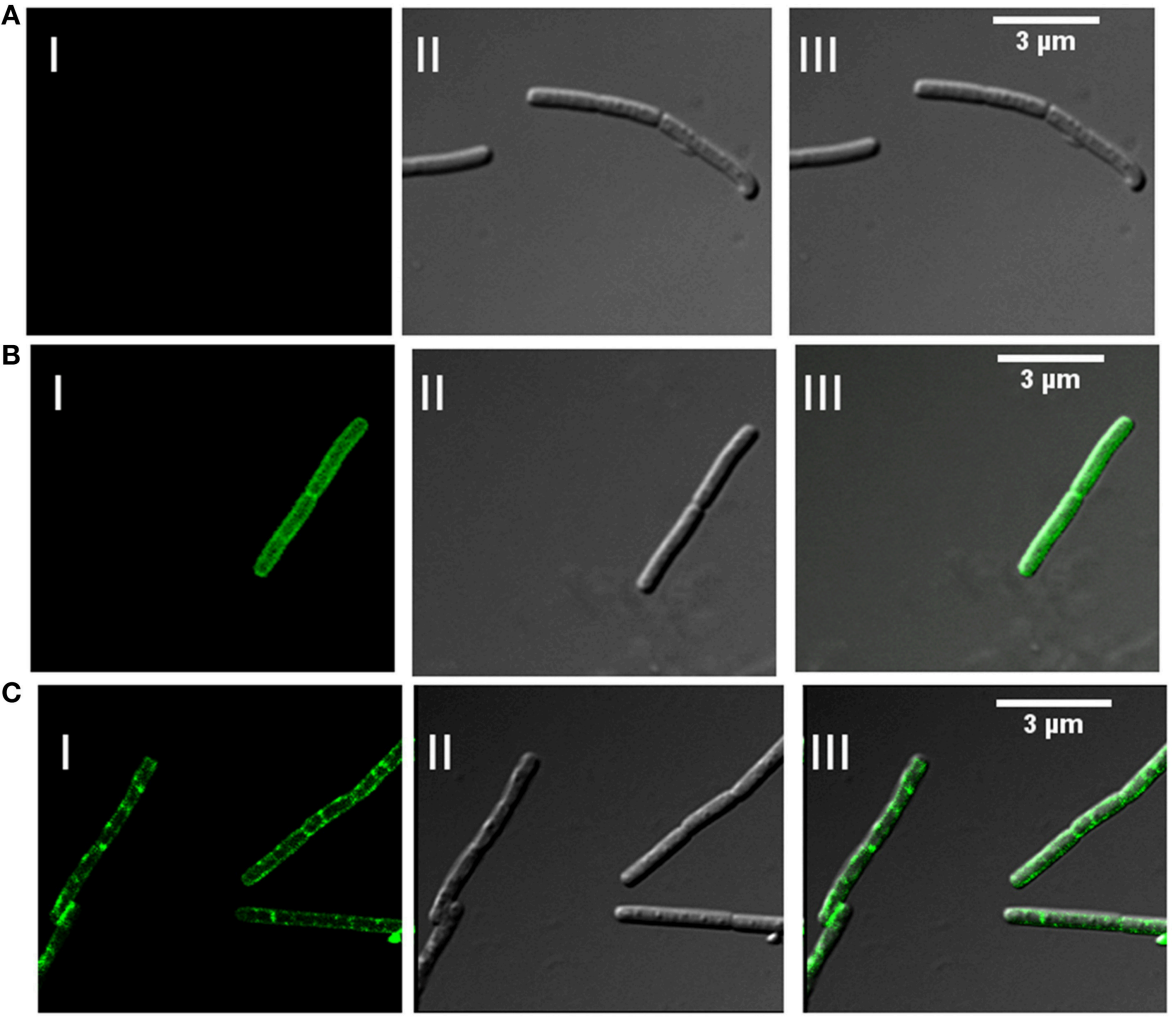
**Heterogeneous distribution of FlotP on membrane of *B. anthracis* by indirect immunofluorescence analysis using Confocal microscopy. (A)** *B. anthracis* cells with pre-immune sera, **(B)** homogenous distribution of GroEL over the surface of bacteria with anti-rGroEL antibody, and **(C)** heterogeneous distribution of FlotP with anti-rFlotP antibody. Images were captured at 100X magnification and zoomed twice for final images. Anti-mouse IgG-FITC antibody was used as secondary antibody. In each Figure, panels I, II, and III represents FITC channel, DIC channel, and superimposition of panels I and II, respectively.

In addition to association with DRM, membrane microdomains provide heterogeneity to the cell membrane (Simons and Vaz, 2004) and associated proteins display punctate distribution. Heterogeneous distribution in specialized membrane microdomains has been observed for many bacterial signaling proteins (Donovan and Bramkamp, 2009). This is supported by the report of punctate and heterogeneous distribution on membrane for Flotillin-like proteins in *B. halodurans* and *B. subtilis* (Donovan and Bramkamp, 2009). In order to determine the distribution pattern of FlotP in *B. anthracis*, we performed indirect immunofluorescence analysis using confocal microscopy. FITC binding was observed as non-uniform green fluorescent patches on the membrane representing the heterogeneous distribution of FlotP (Figure 5C). Anti-GroEL antibody, taken as a negative control showed uniform fluorescence over the entire bacterial surface (Figure 5B) but no such fluorescence was observed with pre-immune sera (Figure 5A). GroEL, a chaperone is known to be distributed uniformly over the cell surface in many bacteria including *B. anthracis* (Hennequin et al., 2001; Sinha and Bhatnagar, 2010). Thus, the above data confirmed the heterogeneous distribution of FlotP on the cell membrane favoring eukaryotic microdomain-like feature on membrane of prokaryotic pathogen. The role of such proteins in activating signaling cascades and receptor activities might be facilitated by their compartmentalization in restricted membrane domains (Troost et al., 2004).

### FlotP is constitutively expressed in *B. anthracis* at all stages of growth

Eukaryotic Flotillin is a highly conserved protein, known to express constitutively in almost all cell types (Babuke and Tikkanen, 2007). Till date this protein has not been reported with any natural mutation (Kokubo et al., 2000). Of note is that changes in mammalian Flotillin-1 expression levels have been found to be associated with Alzheimer's disease (Kokubo et al., 2000) and type 2 diabetes (James et al., 2001).

**Figure 6:**
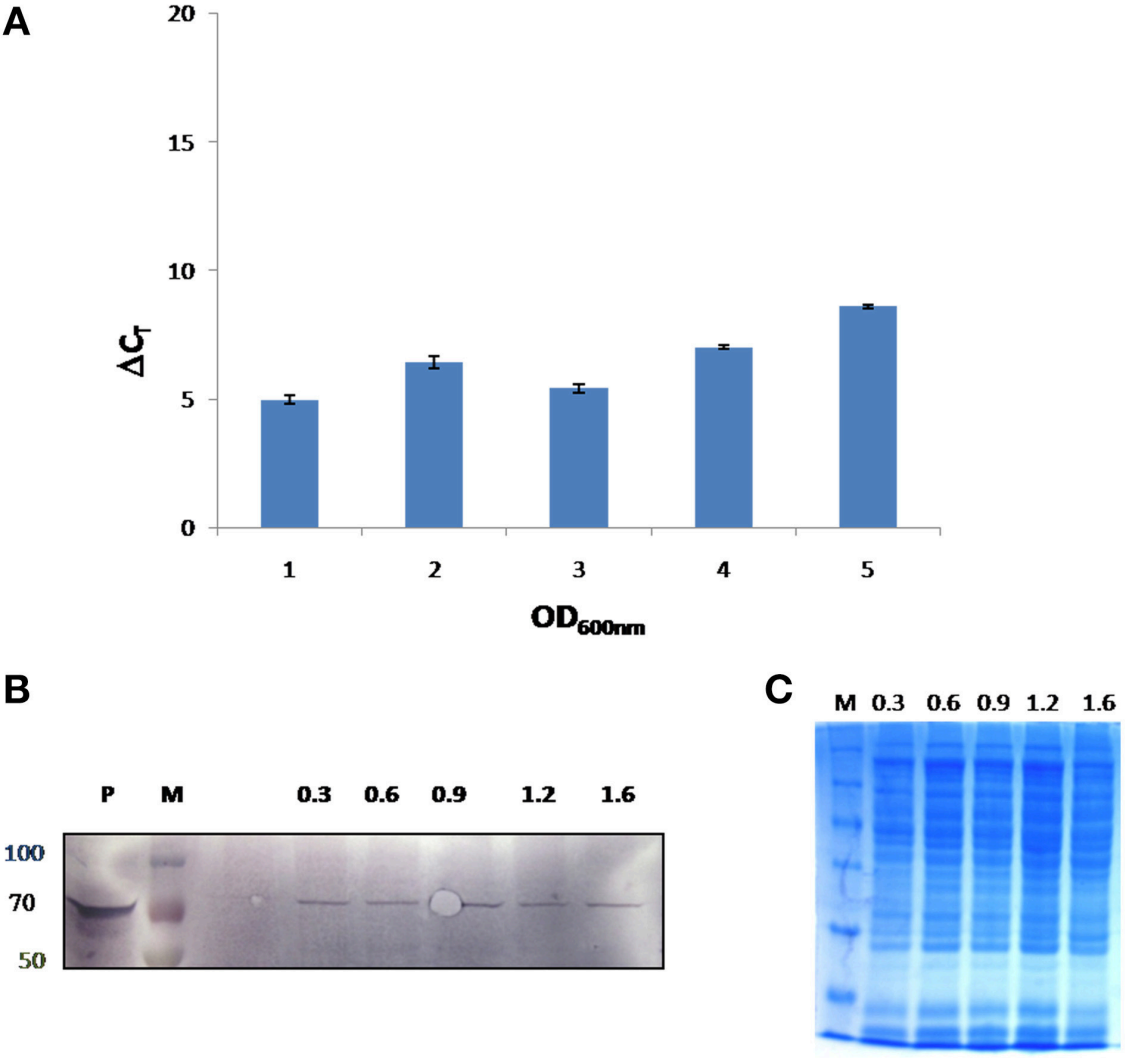
***In vivo* expression of FlotP during different growth phases of *B. anthracis.* (A)** RT-PCR was done for *FlotP* transcript using total RNA of various growth stages. Graph represents normalized Ct value with respect to different stages of growth. Data is a representative of three independent experiments showing similar trend. **(B)** Immunoblotting for *in vivo FlotP* expression in *B. anthracis* at different growth stages. Lanes: P- Purified rFlotP, M- marker, and rest indicate OD_600_ i.e., 0.3, 0.6, 0.9, 1.2, 1.6 for different growth stages taken. **(C)** SDS page showing loaded protein for immunobloting of each OD_600_ level.

In this study, growth stage specific expression of FlotP in *B. anthracis* was analyzed both at protein and transcript levels. For this, different growth stages were selected on the basis of growth curve of *B. anthracis* viz. early exponential, mid exponential, late exponential, early stationary, and stationary phase. FlotP showed constitutive expression at all stages of growth both at mRNA and protein levels as evident from Figure 6. In parallel, we also analyzed the expression of FlotP at protein level in virulent strain of *B. anthracis* also Figure S7 which confirm its presence in most pathogenic form of *B. anthracis*.

**Figure 7:**
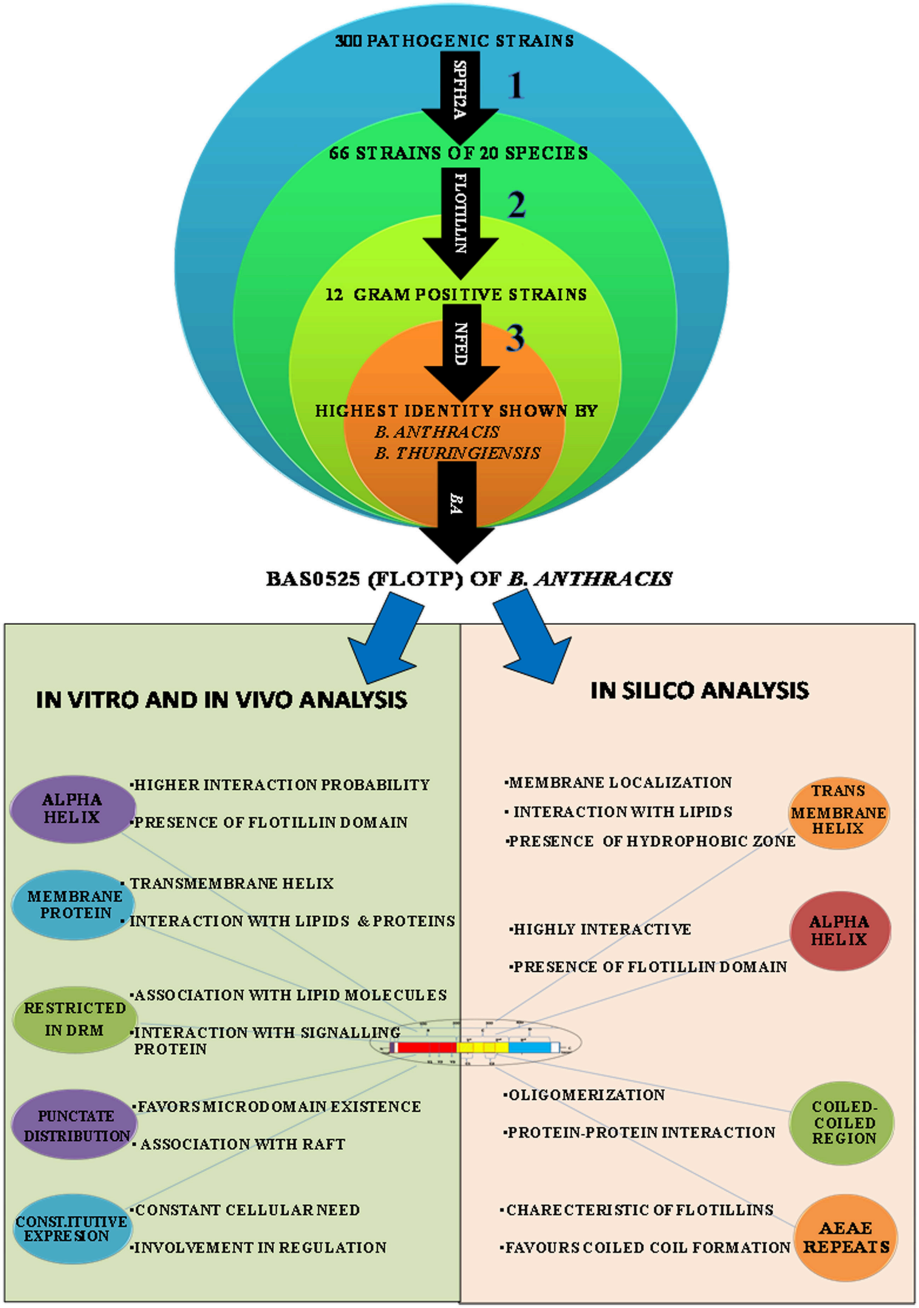
**Graphical representation of the highlights (strategies used at different steps and outcome) of this study**. *In silico, in vitro*, and *in vivo* approaches were employed for characterization of FlotP, an eukaryotic Flotillin homolog. Numericals represent the different steps of study; bold arrows indicate the domains used for the basis of homology search.

In eukaryotes, constitutive expression of Flotillin has been implicated in various crucial cellular processes including insulin signaling, membrane trafficking, phagocytosis, endocytosis, epidermal growth factor receptor signaling, T-lymphocyte activation, cell motility, and transformation (Babuke and Tikkanen, 2007). Constitutive expression of FlotP suggests its critical role in various physiological and cellular processes in bacteria such as communication between cell and environment for activation of signaling pathways (Agarwal et al., 2008; Donovan and Bramkamp, 2009). Similar to eukaryotes, Flotillin homologs in non-pathogenic bacteria are active across the membrane to facilitate interaction and oligomerization of proteins (Gardy et al., 2005; Meile et al., 2006). Membrane microdomains in bacteria harboring flotillin homologs known so far have been found to contain Flotillin-1 homologs associated with signaling and transport proteins (Donovan and Bramkamp, 2009). Moreover, studies have also implicated that these proteins are involved in non-redundant and diverse vital functions related to sporulation, cell shape and motility (López and Kolter, 2010). Therefore, the constitutive expression of this protein may be necessitated by pathogen to fulfill their basic structural and functional attributes, making it a significant target for pre or post infection stage in infectious diseases. A graphical representation of the highlights of this part of the study has been depicted in Figure 7.

### Effect of raft associated lipids biosynthetic pathway inhibitors on *B. anthracis*

In order to validate our hypothesis of raft existence on *B. anthracis* membrane, apart from characterization of FlotP, homolog of raft marker protein Flotillin-1, we extended our study toward the search of raft associated lipid candidate. We carried out our study by analyzing the effect of a raft associated lipid biosynthetic pathway inhibitor, Zaragozic acid (ZA). ZA, a compelling inhibitor of squalene synthase, blocks sterol biosynthesis specifically cholesterol synthesis in mammals and ergosterol synthesis in fungi which are the well-known raft associated lipid moieties (Bergstrom et al., 1995; de Souza and Rodrigues, 2009). Although, there is no direct evidence of presence of sterols in bacteria, the foremost report of existence of lipid raft on *B. subtilis* membrane showed the significant effect of ZA (López and Kolter, 2010) on its physiology. Its effect on lipid raft integrity and thus Flotillin localization suggest the occurrence of similar kind of moieties in bacteria also.

To corroborate the presence of sterol derivative in pathogen we analyzed effect of ZA on several pathophysiological attributes of *B. anthracis* like growth, morphology, membrane fluidity and toxin secretion.

#### ZA slows down the growth of *B. anthracis*

**Figure 8:**
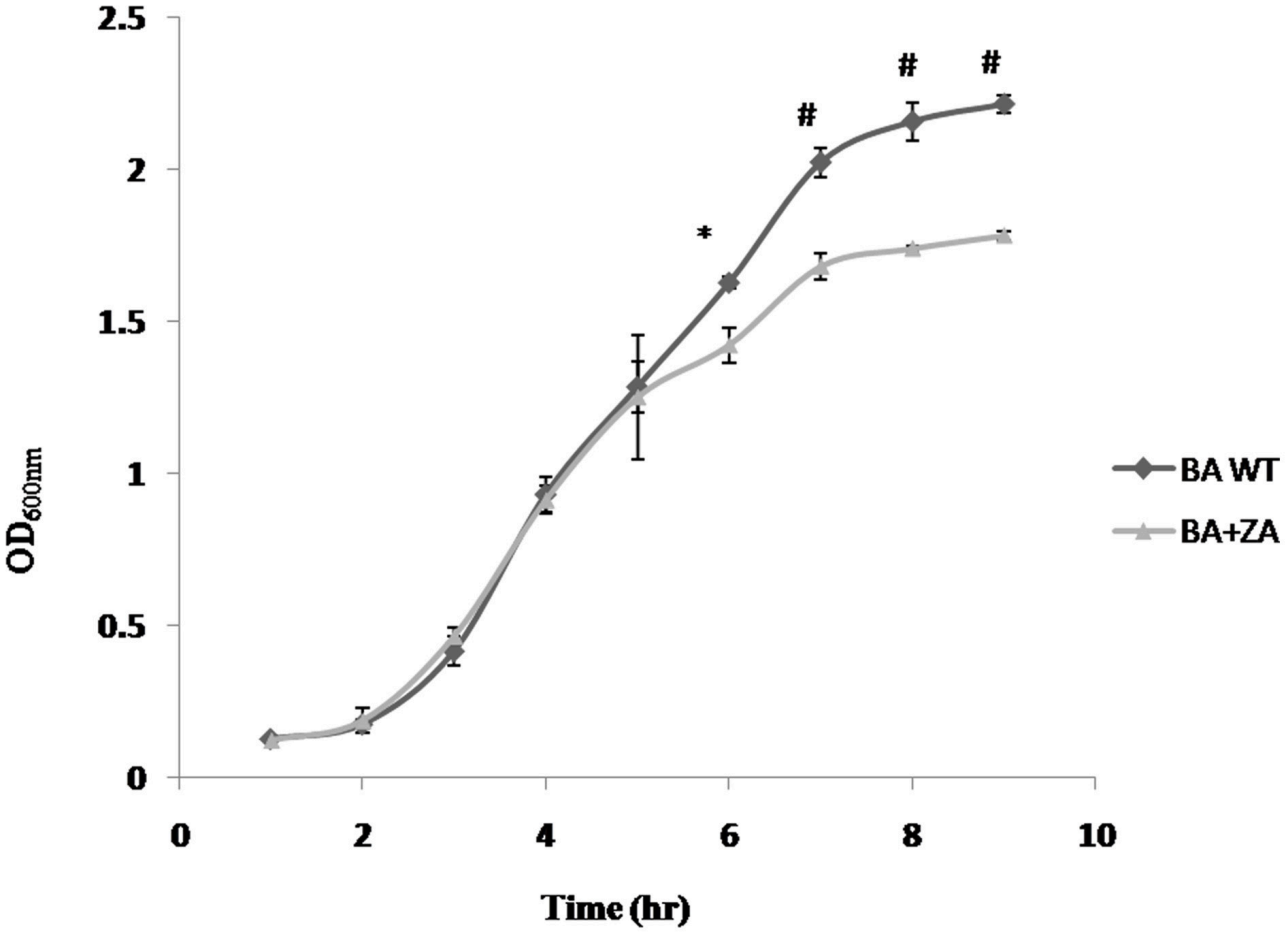
**Effect of ZA on growth of *B. anthracis***. Data is standard mean ± SME of three independent experiments. ^*^indicate *p* ≤ 0.05 and ^#^indicates *p* ≤ 0.001.

To determine the effect of ZA on growth of bacteria, *B. anthracis* Sterne was inoculated in BHI media containing 1% NaHCO_3_ in presence or absence of ZA and the growth was examined periodically. As shown in Figure 8, an insignificant variation was observed for first few hours (till mid log phase). However, a drastic reduction in growth was observed at stationary phase (*p* < 0.01).

The observation is completely different from what has been reported in *B. subtilis* where presence of ZA did not affect the growth (López and Kolter, 2010). However, in yeast (Machida et al., 1999; Hornby et al., 2003) and mammals (Daicho et al., 2007; Joo and Jetten, 2010), ZA has been found to decrease the growth rate by increasing the level of farnesol which affect growth by altering cell cycle signaling mechanism and by apoptosis, respectively. Moreover, in some prokaryotes like *S. aureus*, farnesol has also been seen to have a negative effect on growth of the cells through some undefined interaction with the cell membrane (Jabra-Rizk et al., 2006) In addition, some studies also suggest that even a little deviation in the membrane integrity of the cell affects rate of replication causing reduction in growth rate (Firshein, 1989; Firshein and Kim, 1997; Nordström, 2003). Thus, we may hypothesize that the above observation may be due to some possible change in the *B. anthracis* membrane due to the presence of ZA that needs to be further explored.

#### ZA increases the cell dimension

**Figure 9:**
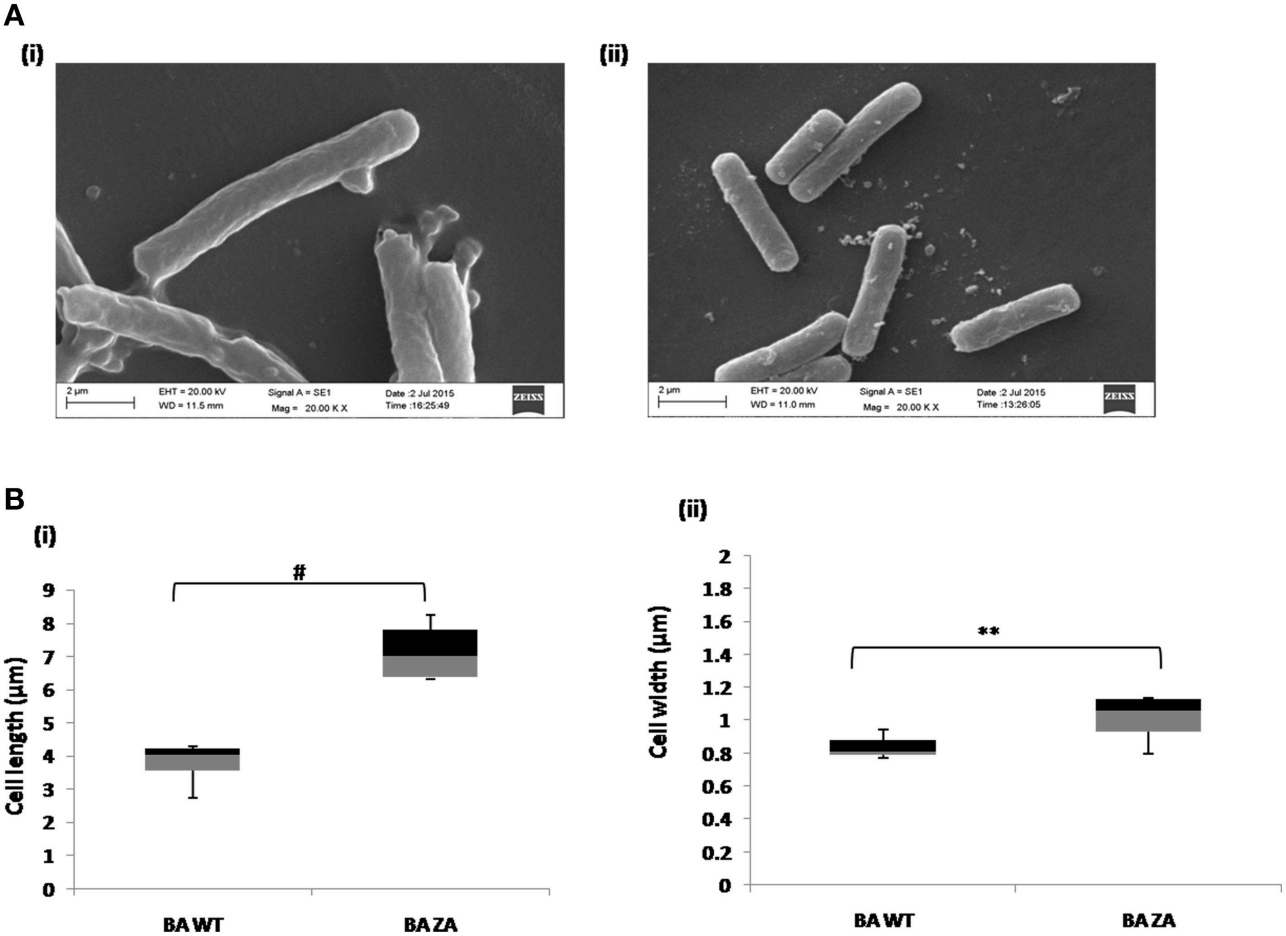
**Effect of ZA on morphology of *B. anthracis.* (A)** Stationary phase cells from culture grown with **(i)** or without ZA **(ii)** were used for SEM analysis. **(B)** Box-and-whiskers plot to represent the cell dimensions where maximum and minimum size of population, median, and 25th and 75th population percentile is represented by whiskers, center bar, and each box, respectively. **(i)** cell length, **(ii)** cell width. ^**^indicates *p* ≤ 0.01 and ^#^indicates *p* ≤ 0.001.

As per our above observations, any alteration in the membrane integrity affects the growth by affecting rate of replication and hence, it must also affect the cell morphology. Considering this, morphology of ZA treated as well as untreated cells were analyzed by SEM (Figure 9A). Interestingly, cells in presence of ZA were found to increase the mean cell length (Figure 9Bi) as well as width (Figure 9Bii) significantly by ~2 times (*p* < 0.001) and ~1.2 times (*p* < 0.01), respectively, as compared to untreated cells. Table 3 indicated the comparison of mean cell dimensions.

**Table 3:** **The cell dimensions (μm) of *B. anthracis* cells in presence (BA+ZA) or absence (BA WT) of ZA**.

	**BA WT**		**BA**+**ZA**	
	**Length**	**Width**	**Length**	**Width**
Mean	3.7625	0.836	7.148	1.017
SD	0.7	0.063	0.863	0.139

Mean and standard deviation of nearly 10 cells from three independent experiments are shown.

Moreover, in yeast also ZA has been found to affect the cell cycle by inhibiting replication (Machida et al., 1999) thus we may hypothesize that the variation observed in cell size might be due to down regulation of replication which might be due to the deviation in organized membrane in presence of ZA. However, detailed study has to be done which is beyond the scope of this study.

#### ZA renders membrane of *B. anthracis* more fluid

Fluidity or rigidity are the physical state parameters of membrane (Barrera et al., 2012), which is strongly influenced by raft rigidity that in turn depend on the concentration of sterol derivatives in it (Mattson, 2005). Cells adapt to the changes in environment through modulation of membrane properties and lipid composition resulting in altered membrane fluidity (Barrera et al., 2012). Cell membrane serves as a barrier for entry or exit of biomolecules across the cells and intrinsic (inherent) or acquired cellular changes due to alteration in structure may accompany the cells to modulate the pathogenicity and virulence of the organism (Diakogiannis et al., 2013). In order to determine the effects of endogenous sterol derivatives on membrane fluidity, the biophysical properties of the *B. anthracis* membrane was accessed in presence of ZA using fluorescence polarization and TRFS.

**Figure 10:**
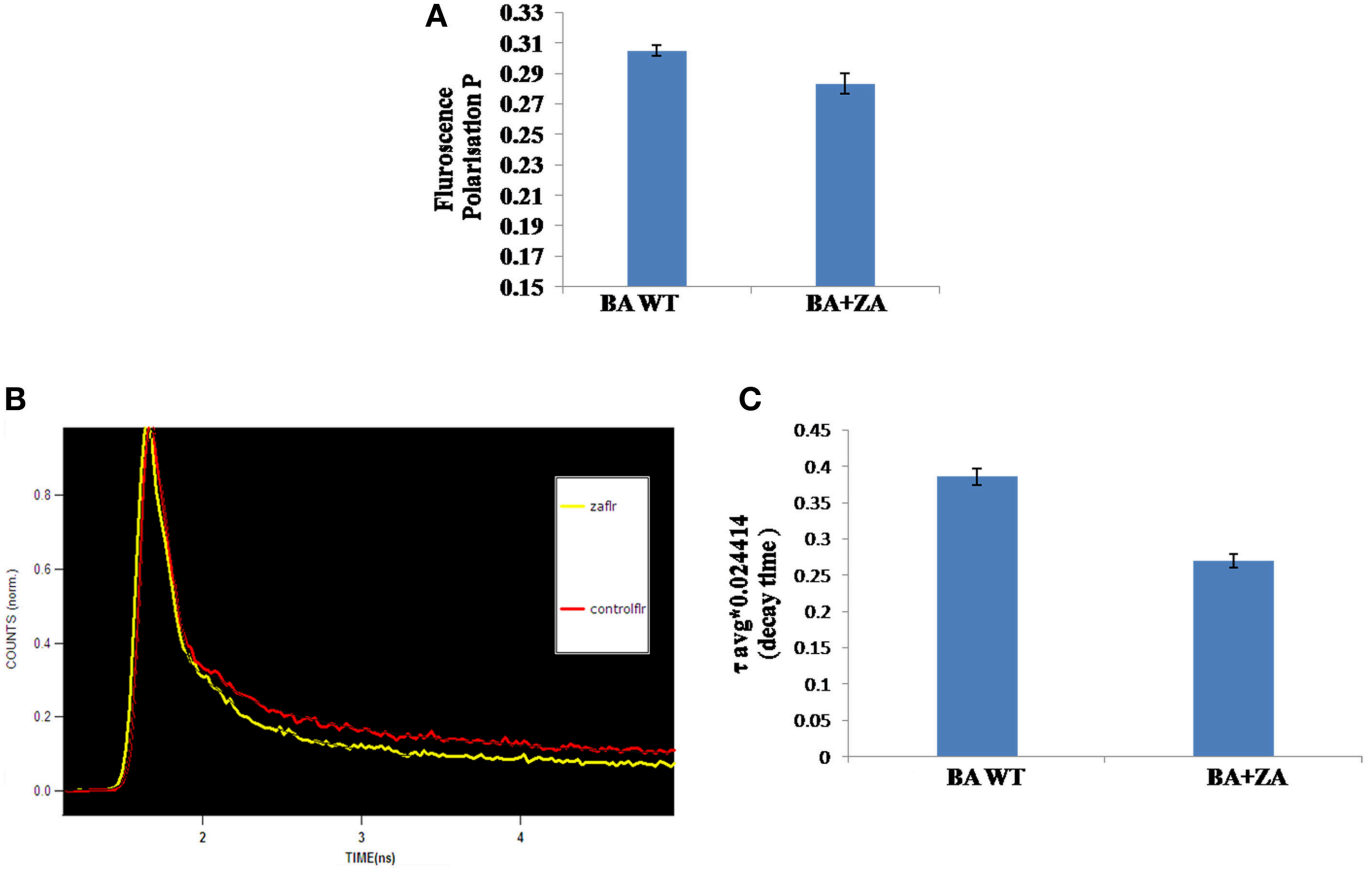
**Effect of ZA on membrane fluidity of *B. anthracis.* (A)** Mean fluorescence polarization “*p*” values (inversely propotional to membrane fluidity) of *B. anthracis* grown in absence (control) and presence of ZA. **(B)** Decay curve for DPH labeled *B. anthracis* grown with or without ZA. **(C)** Time dependent Fluorescence decay (with fits) in linear scale with respect to time in nanoseconds of DPH in the ZA treated and untreated cells.

Steady state fluorescence polarization gives an average measure of the membrane fluidity/rigidity of all the cells. This study revealed a significant change in the fluorescence polarization in *B. anthracis* in presence of ZA in the media (Figure 10A). The control cells grown without ZA had a higher membrane order and therefore, displayed higher fluorescence polarization values i.e., more rigid membrane than the ZA treated cells.

Sterol rich membrane microdomains have been reported to play a crucial role in membrane structure, organization, and function in eukaryotes. As already stated, fluorescence polarization is inversely proportional to membrane fluidity and decrease in fluorescence polarization values are typically due to an increase in rotational mobility of the fluorophore, which is influenced by packing of sterols and fatty acyl chains.

In addition, to further confirm the above observed result, membrane fluidity was also measured by TRFS which measure the time resolved fluorescence decay of fluorescent molecules (DPH) within a specific lipid environment and thus gives a measure of microviscosity of cells. Fluorescence lifetime is a reliable indicator of polarity changes in local environment of the DPH. Life time of DPH has been found to be reduced in presence of water in its immediate environment (Arora et al., 2004).

The data revealed that decay time for DPH in Zaragozic acid treated cells are lower than the decay time in the untreated (control) bacterial cells by 31.6% (Figures 10B,C, Table 4). Water penetration may be more due to lesser rigidifying effect of sterol/other raft associated lipids as revealed by fluorescence polarization. These decay patterns provided the observed changes in the cellular microenvironment of the respective membrane after treatment with various inhibitors

**Table 4:** **Fluorescence lifetime decay of DPH in nanoseconds (ns) at mid log phase untreated cells (control) and *B. anthracis* cells treated with ZA**.

**S.no**.	**Inhibitors**	**Decay time (ns)**	**% change in decay time**
1	Control	0.375 ± 0.01	–
2	Zaragozic acid	0.259 ± 0.01	31.6

The data obtained from TRFS validated the experiments results of steady state fluorescence polarization. The increase in fluidity for membrane of bacterial cells grown in the presence of zaragozic acid corroborates with the decrease in decay time of DPH in cells grown with these inhibitors. Decay of DPH becomes faster in fluid membrane due to decrease in microviscosity.

These results indicated the possibility of existence of sterol surrogates or lipid candidates in bacterial microdomains similar to eukaryotic sterol asssociated with lipid rafts which has been seen to be affected by fungal sterol biosynthetic pathways inhibitors and thus, treatment with such inhibitors possibly lead to altered composition of such lipid molecules resulting in loose packing of membranes and enhanced membrane fluidity. The observed results may be due to differences in the levels of compaction of cell membrane between untreated and ZA treated bacterial cells clearly suggesting the possibility of existence of sterol surrogates, other lipid candidates and raft associated membrane proteins.

#### ZA decreases toxin secretion by *B. anthracis*, thus has potential to reduce virulence

**Figure 11:**
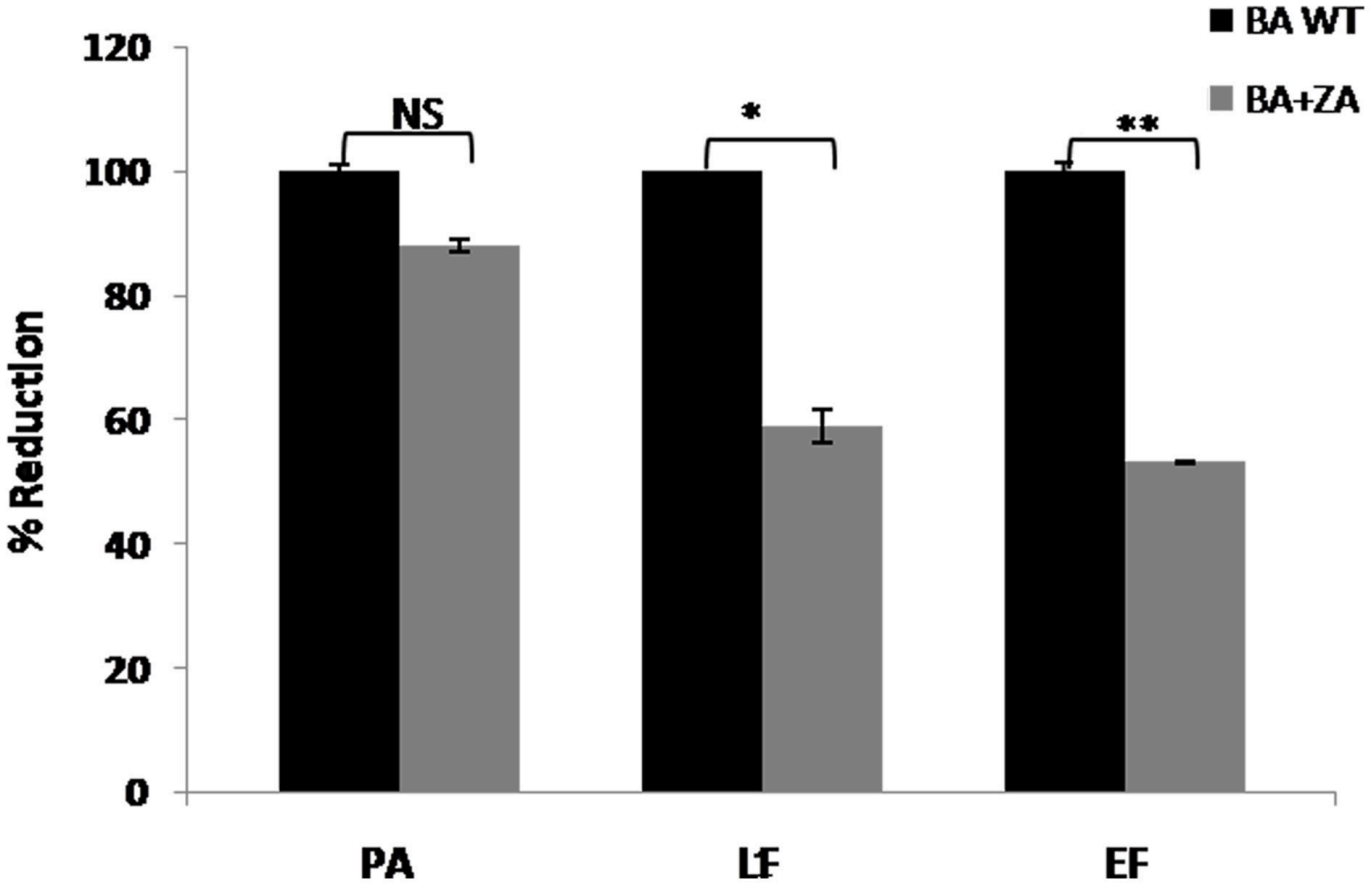
**Effect of ZA on toxin production by *B. anthracis.*** Quantitation of major virulence factors (PA, LF, and EF) was done by ELISA. Bars represent the percent reduction of *B. anthracis* treated with ZA (BA+ZA) with respect to untreated cells (BA WT). NS, non-significant; ^*^p < 0.05 and ^**^p < 0.01.

Secretion of virulence factors (PA, Protective antigen; LF, Lethal factor; EF, Edema factor) by *B. anthracis* in extracellular milieu is a major contributing virulence factor leading to anthrax. Though the mechanism of their secretion is still unknown, a recent report suggested the secretion of these factors in the form of vesicles involving variety of lipids on vesicular membrane (Rivera et al., 2010). Considering the fact that lipid raft like entities possess a large variety of transport or secretory proteins, we were interested in finding whether there is any relation between raft and toxin secretion in *B. anthracis.* Therefore, we carried out this part of study by determining the effect of ZA on toxin secretion. For this, media supernatants of *B. anthracis* grown with or without ZA was collected and quantitaion of toxin components was done by ELISA using monoclonal antibodies against PA, LF and EF. In our analysis, ZA was found to decrease significantly the secretion of LF and EF by ~41% (*p* < 0.05) and ~47% (*p* < 0.01), respectively whereas, no significant change was observed in PA (*p* > 0.05; Figure 11).

Although the exact role of ZA in secretion of toxin components is yet to be established but our data suggest the involvement of lipid raft in this crucial physiological process and thus, pathogenesis of *B. anthracis.* Therefore, we may propose that targeting lipid raft or use of anti-raft compounds may provide alternate treatment strategy against anthrax or other raft harboring pathogens also.

### ZA affects expression and localization of FlotP

**Figure 12:**
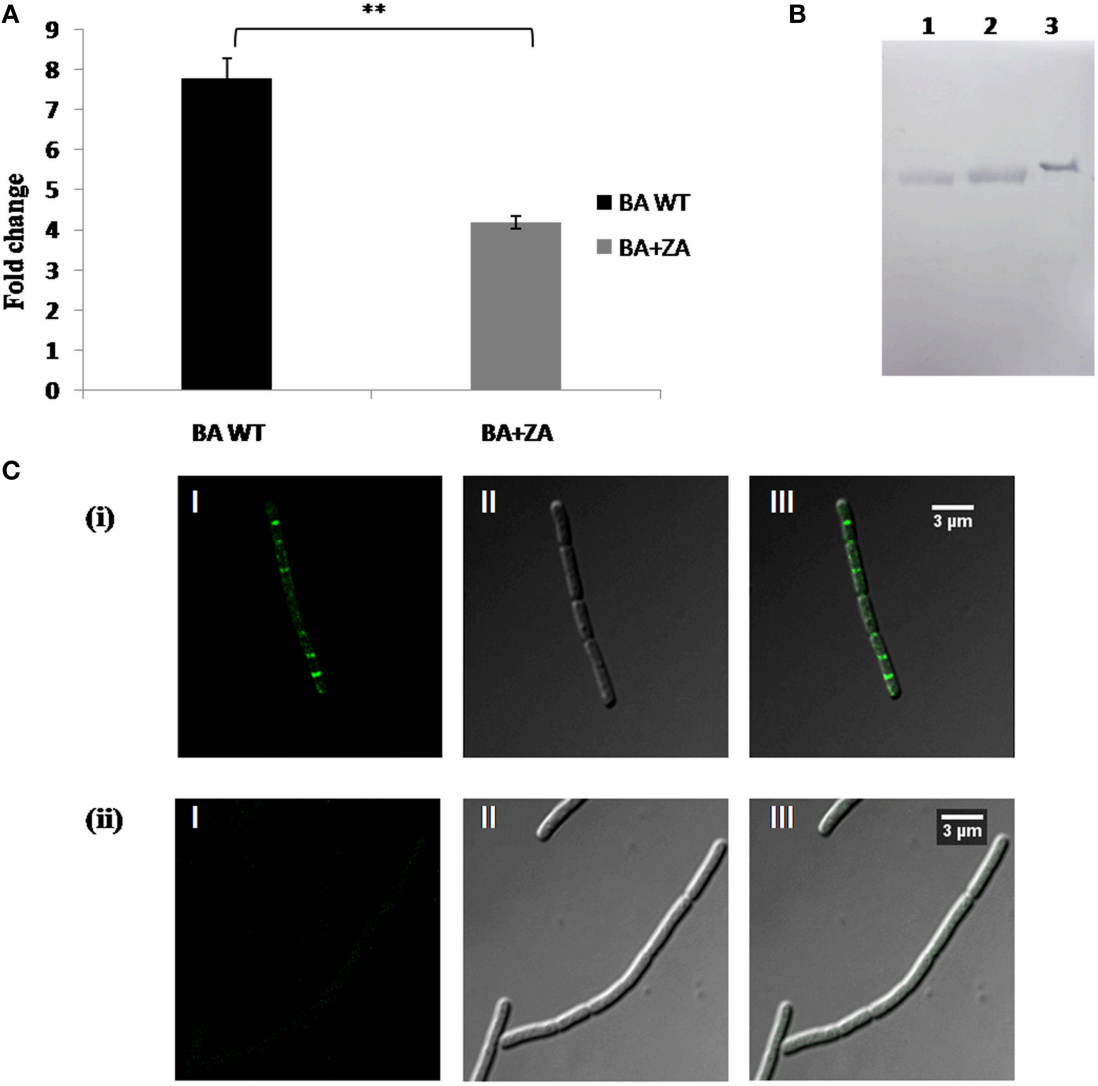
**Effect of ZA on FlotP expression *in vivo.* (A)** Data represents the fold change in *flotP* expression at mRNA level by qRT–PCR taking *dna gyrase* as a constitutively expressed gene for normalization of C_T_ values. The experiment was repeated two times biologically with three technical replicates. Data shown here is mean ± SME of one of the two independent biological experiment. ^**^indicates *p* ≤ 0.01. **(B)** Expression analysis of FlotP at protein level by immunoblotting. Lanes: 1, Total cell lysate of ZA treated *B. anthracis* cells; 2, total cell lysate of ZA untreated cells; 3, purified rFlotP. **(C)** Immunofluorescence analysis of FlotP localization on *B. anthracis* cell surface using confocal microscopy; BA WT **(i)**, BA+ZA **(ii)**. In each figure, panels I, II, and III represents FITC channel, DIC channel, and superimposition of panels I and II, respectively.

Our study revealed the membrane localization of FlotP as well as the existence of some unknown sterol derivative on *B. anthracis* cell surface. To check whether there is any link between FlotP and anonymous lipid moieties, the effect of ZA on FlotP expression was analyzed both at transcript as well as protein level. qRT-PCR analysis showed a significant decrease of approximately Two-fold in *flotP* transcript level in ZA treated cells (Figure 12A). Interestingly, even at protein level, expression of FlotP was found to be decreased by ~1.8-folds as observed by immunoblotting (Figure 12B). These observations suggest the possibility of some regulatory mechanism to coordinate expression of FlotP according to change in the endogenous availability of sterol derivatives. Thus, we may hypothesize that certain genetic coordination might be existing between raft protein FlotP and sterol kind of lipid moieties in addition to their physical association in the raft of cell.

In addition, immunofluorescence analysis revealed disruption in the localization of FlotP on *B. anthracis* cell surface in the presence of ZA (Figure 12C). The result was in consistence with *B. subtilis* where continuous treatment of ZA lead to complete abrogation of enzyme involved in the synthesis of anonymous lipid moiety which in turn disturbed the localization of Flotillin homolog.

Thus, these observations indicated the association of FlotP with some unknown sterol derivatives to form raft like entity. This is the preliminary report about the existence of raft like features in *B. anthracis.* Therefore, the proper mechanism of interaction needs to be explored in order to answer unsolved queries of raft in *B. anthracis.*

## Conclusion

In this study, we have done for the first time, a detailed investigation on the existence of raft like feature in any PB by characterizing it in *B. anthracis.* Homolog of Flotillin-1, FlotP of *B. anthracis*, showed similarity in genetic organization, domain architecture as well as distribution pattern with other known RMPs. Constitutive expression of FlotP indicates its possible critical role which might range from basic bacterial metabolism to virulence via regulatory or signaling pathways. Moreover, studies done with anti-raft agent ZA revealed its significant effect on patho-physiology of BA such as growth, morphology, membrane fluidity, toxin secretion, FlotP expression as well as its distribution over cell surface etc. Thus, it may be concluded that lipid rafts have the potential to serve as a new target for controlling bacterial virulence and infections. Lipid raft targeted drugs are likely to have broad spectrum activity, lower microbial resistance and minimal host cell toxicity, thus making them excellent candidates for future use in biomedical applications to address challenges of bacterial infections. Further exploration of the candidate lipid molecule involved in prokaryotic raft formation may prove assistive in identifying other raft harboring pathogens. The specificity of lipid as well as protein of the pathogenic raft would lead to the development of potential anti-infectious agent.

## Author contributions

VS designed, performed and analyzed the experiments; SA performed and analyzed the experiments; DS performed few experiments; VS, SA, TP, RB wrote paper, RB contributed reagents, materials and instruments.

### Conflict of interest statement

The authors declare that the research was conducted in the absence of any commercial or financial relationships that could be construed as a potential conflict of interest.

## Abbreviations

SPFH: Stomatin, Prohibitin, Flotillin, and HflK/C protein super family
DRM: Detergent Resistant Membrane
DSM: Detergent Sensitive Membrane
CDS: Circular Dichroism Spectroscopy.
