# Supplementary material for: Identification of Novel Raft Marker Protein, FlotP in Bacillus anthracis
Source: Front Microbiol. 2016 Feb 17;7:169. doi: 10.3389/fmicb.2016.00169 (PMC4756111; doi:10.3389/fmicb.2016.00169)

Supplementary file S1:

Phylogenetic tree showing all pathogens harboring SPFH2a super family protein.

Initial 3 or 4 letters are code of species followed by code of gene encoding SPFH2a protein.

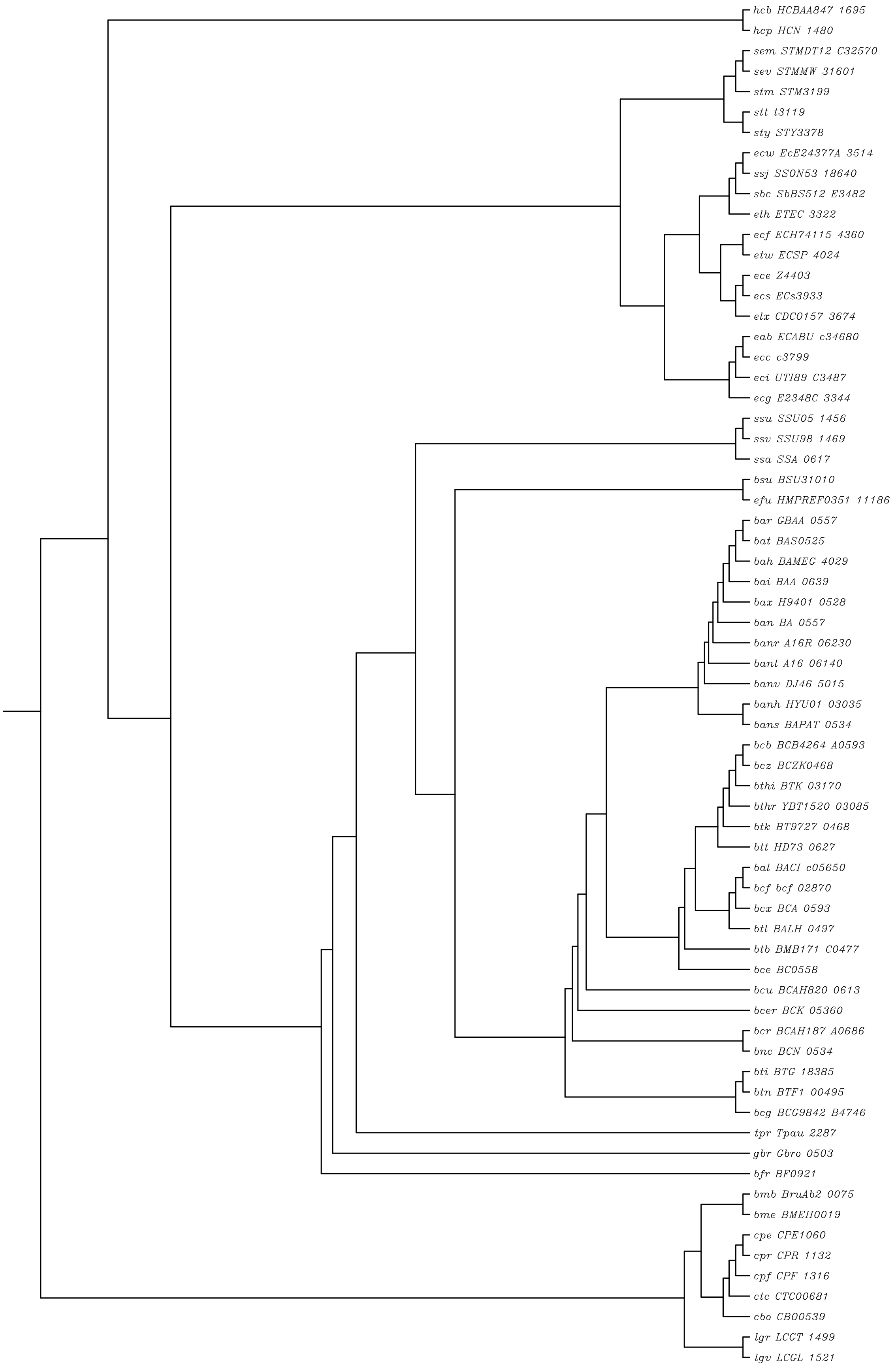

Supplement: Supplementary file 1 [file DataSheet1.PDF]
