# Supplementary material for: Identification of Novel Raft Marker Protein, FlotP in Bacillus anthracis
Source: Front Microbiol. 2016 Feb 17;7:169. doi: 10.3389/fmicb.2016.00169 (PMC4756111; doi:10.3389/fmicb.2016.00169)

**Supplementary File S2: List of 66 pathogens harboring proteins with SPFH2a domain taking *B. subtilis* as reference.**

**(A)** Name of organism with the code and length protein. **(B)** SPFH2a domain alignment of all pathogens with *B. subtilis* SPFH2a as a reference. Red indicates the similarity; Blue indicates mismatch.

| **Sequence No.** | **Species name** | **Protein code** | **Length of protein (No. of amino acids)** |
| --- | --- | --- | --- |
| **1** | *Bacillus subtilis* | bsu_BSU31010 | 509 |
| **2** | *Enterococcus faecalis* | efu_HMPREF0351_11186 | 499 |
| **3** | *Bacillus cereus* | bcer_BCK_05360 | 526 |
| **4** | *Bacillus cereus biovar anthracis CI* | bal_BACI_c05650 | 524 |
| **5** | *Bacillus cereus F837/76* | bcf_bcf_02870 | 524 |
| **6** | *Bacillus cereus AH187* | bcr_BCAH187_A0686 | 524 |
| **7** | *Bacillus cereus 03BB102* | bcx_BCA_0593 | 524 |
| **8** | *Bacillus cereus NC7401* | bnc_BCN_0534 | 524 |
| **9** | *Bacillus thuringiensis Al Hakam* | btl_BALH_0497 | 524 |
| **10** | *Bacillus cereus AH820* | bcu_BCAH820_0613 | 526 |
| **11** | *Bacillus anthracis HYU01* | banh_HYU01_03035 | 526 |
| **12** | *Bacillus anthracis SVA11* | bans_BAPAT_0534 | 526 |
| **13** | *Bacillus cereus B4264* | bcb_BCB4264_A0593 | 524 |
| **14** | *Bacillus cereus ZK* | bcz_BCZK0468 | 524 |
| **15** | *Bacillus thuringiensis serovar kurstaki HD-1* | bthi_BTK_03170 | 524 |
| **16** | *Bacillus thuringiensis serovar kurstaki YBT-1520* | bthr_YBT1520_03085 | 524 |
| **17** | *Bacillus thuringiensis 97-27* | btk_BT9727_0468 | 524 |
| **18** | *Bacillus thuringiensis serovar kurstaki HD73* | btt_HD73_0627 | 524 |
| **19** | *Bacillus anthracis CDC 684* | bah_BAMEG_4029 | 526 |
| **20** | *Bacillus anthracis A0248* | bai_BAA_0639 | 526 |
| **21** | *Bacillus anthracis Ames* | ban_BA_0557 | 526 |
| **22** | *Bacillus anthracis A16R* | banr_A16R_06230 | 526 |
| **23** | *Bacillus anthracis A16* | bant_A16_06140 | 526 |
| **24** | *Bacillus anthracis Vollum* | banv_DJ46_5015 | 526 |
| **25** | *Bacillus anthracis Ames 0581* | bar_GBAA_0557 | 526 |
| **26** | *Bacillus anthracis Sterne* | bat_BAS0525 | 526 |
| **27** | *Bacillus anthracis H9401* | bax_H9401_0528 | 526 |
| **28** | *Bacillus cereus ATCC 14579* | bce_BC0558 | 524 |
| **29** | *Bacillus thuringiensis BMB171* | btb_BMB171_C0477 | 524 |
| **30** | *Bacillus cereus G9842* | bcg_BCG9842_B4746 | 524 |
| **31** | *Bacillus thuringiensis HD-771* | bti_BTG_18385 | 524 |
| **32** | *Bacillus thuringiensis HD-789* | btn_BTF1_00495 | 524 |
| **33** | *Streptococcus sanguinis* | ssa_SSA_0617 | 492 |
| **34** | *Streptococcus suis 05ZYH33* | ssu_SSU05_1456 | 489 |
| **35** | *Streptococcus suis 98HAH33* | ssv_SSU98_1469 | 489 |
| **36** | *Tsukamurella paurometabola* | tpr_Tpau_2287 | 467 |
| **37** | *Bacteroides fragilis YCH46* | bfr_BF0921 | 541 |
| **38** | *Gordonia bronchialis* | gbr_Gbro_0503 | 370 |
| **39** | *Salmonella enterica subsp. enterica serovar Typhimurium T000240* | sem_STMDT12_C32570 | 559 |
| **40** | *Salmonella enterica subsp. enterica serovar Typhimurium D23580* | sev_STMMW_31601 | 559 |
| **41** | *Salmonella enterica subsp. enterica serovar Typhimurium LT2* | stm_STM3199 | 559 |
| **42** | *Escherichia coli E24377A (ETEC)* | ecw_EcE24377A_3514 | 553 |
| **43** | *Shigella sonnei 53G* | ssj_SSON53_18640 | 553 |
| **44** | *Salmonella enterica subsp. enterica serovar Typhi Ty2* | stt_t3119 | 559 |
| **45** | *Salmonella enterica subsp. enterica serovar Typhi CT18* | sty_STY3378 | 559 |
| **46** | *Shigella boydii CDC 3083-94* | sbc_SbBS512_E3482 | 542 |
| **47** | *Escherichia coli O78:H11:K80 H10407 (ETEC)* | elh_ETEC_3322 | 553 |
| **48** | *Escherichia coli O157:H7 EDL933 (EHEC)* | ece_Z4403 | 553 |
| **49** | *Escherichia coli O157:H7 Sakai (EHEC)* | ecs_ECs3933 | 553 |
| **50** | *Escherichia coli O157:H7 Xuzhou21 (EHEC)* | elx_CDCO157_3674 | 553 |
| **51** | *Escherichia coli O157:H7 EC4115 (EHEC)* | ecf_ECH74115_4360 | 553 |
| **52** | *Escherichia coli O127:H6 E2348/69 (EPEC)* | ecg_E2348C_3344 | 553 |
| **53** | *Escherichia coli O157:H7 TW14359 (EHEC)* | etw_ECSP_4024 | 553 |
| **54** | *Escherichia coli ABU 83972* | eab_ECABU_c34680 | 553 |
| **55** | *Escherichia coli O6:K2:H1 CFT073 (UPEC)* | ecc_c3799 | 553 |
| **56** | *Escherichia coli O18:K1:H7 UTI89 (UPEC)* | eci_UTI89_C3487 | 553 |
| **57** | *Helicobacter cinaedi ATCC BAA-847* | hcb_HCBAA847_1695 | 466 |
| **58** | *Helicobacter cinaedi PAGU611* | hcp_HCN_1480 | 319 |
| **59** | *Brucella abortus 9-941* | bmb_BruAb2_0075 | 328 |
| **60** | *Brucella melitensis bv. 1 16M* | bme_BMEII0019 | 328 |
| **61** | *Clostridium perfringens 13* | cpe_CPE1060 | 316 |
| **62** | *Clostridium perfringens SM101* | cpr_CPR_1132 | 316 |
| **63** | *Clostridium perfringens ATCC 13124* | cpf_CPF_1316 | 316 |
| **64** | *Clostridium tetani E88* | ctc_CTC00681 | 313 |
| **65** | *Clostridium botulinum A ATCC 3502* | cbo_CBO0539 | 331 |
| **66** | *Lactococcus garvieae ATCC 49156* | lgr_LCGT_1499 | 297 |
| **67** | *Lactococcus garvieae Lg2* | lgv_LCGL_1521 | 297 |


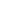


**(B)**


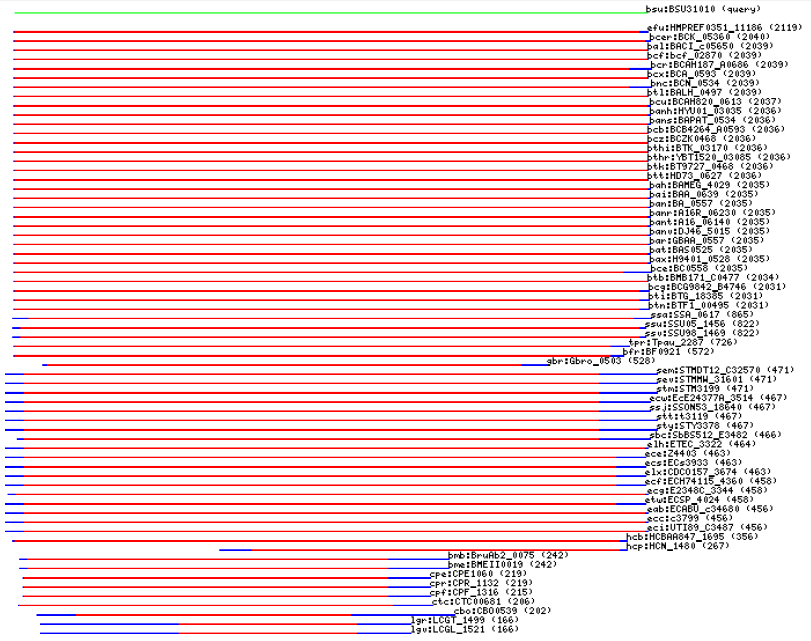

Supplement: Supplementary file 2 [file DataSheet2.DOCX]
