# Supplementary material for: Identification of Novel Raft Marker Protein, FlotP in Bacillus anthracis
Source: Front Microbiol. 2016 Feb 17;7:169. doi: 10.3389/fmicb.2016.00169 (PMC4756111; doi:10.3389/fmicb.2016.00169)
Supplement: Supplementary file 3 [file Presentation1.PPT]

## Slide 1
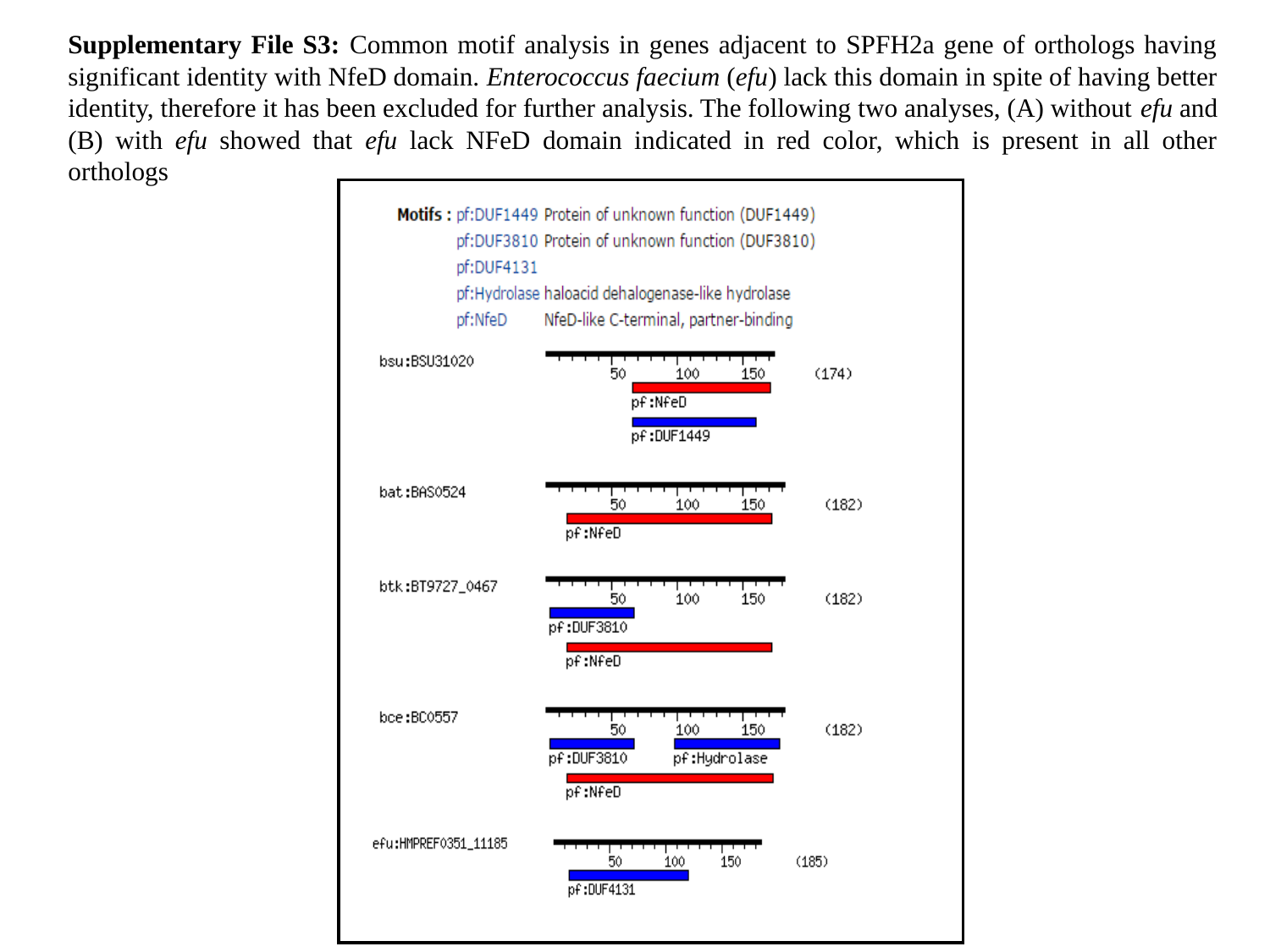

Supplementary File S3: Common motif analysis in genes adjacent to SPFH2a gene of orthologs having significant identity with NfeD domain. Enterococcus faecium (efu) lack this domain in spite of having better identity, therefore it has been excluded for further analysis. The following two analyses, (A) without efu and (B) with efu showed that efu lack NFeD domain indicated in red color, which is present in all other orthologs
