# Supplementary material for: Identification of Novel Raft Marker Protein, FlotP in Bacillus anthracis
Source: Front Microbiol. 2016 Feb 17;7:169. doi: 10.3389/fmicb.2016.00169 (PMC4756111; doi:10.3389/fmicb.2016.00169)
Supplement: Supplementary file 4 [file Presentation2.PPTX]

## Slide 1
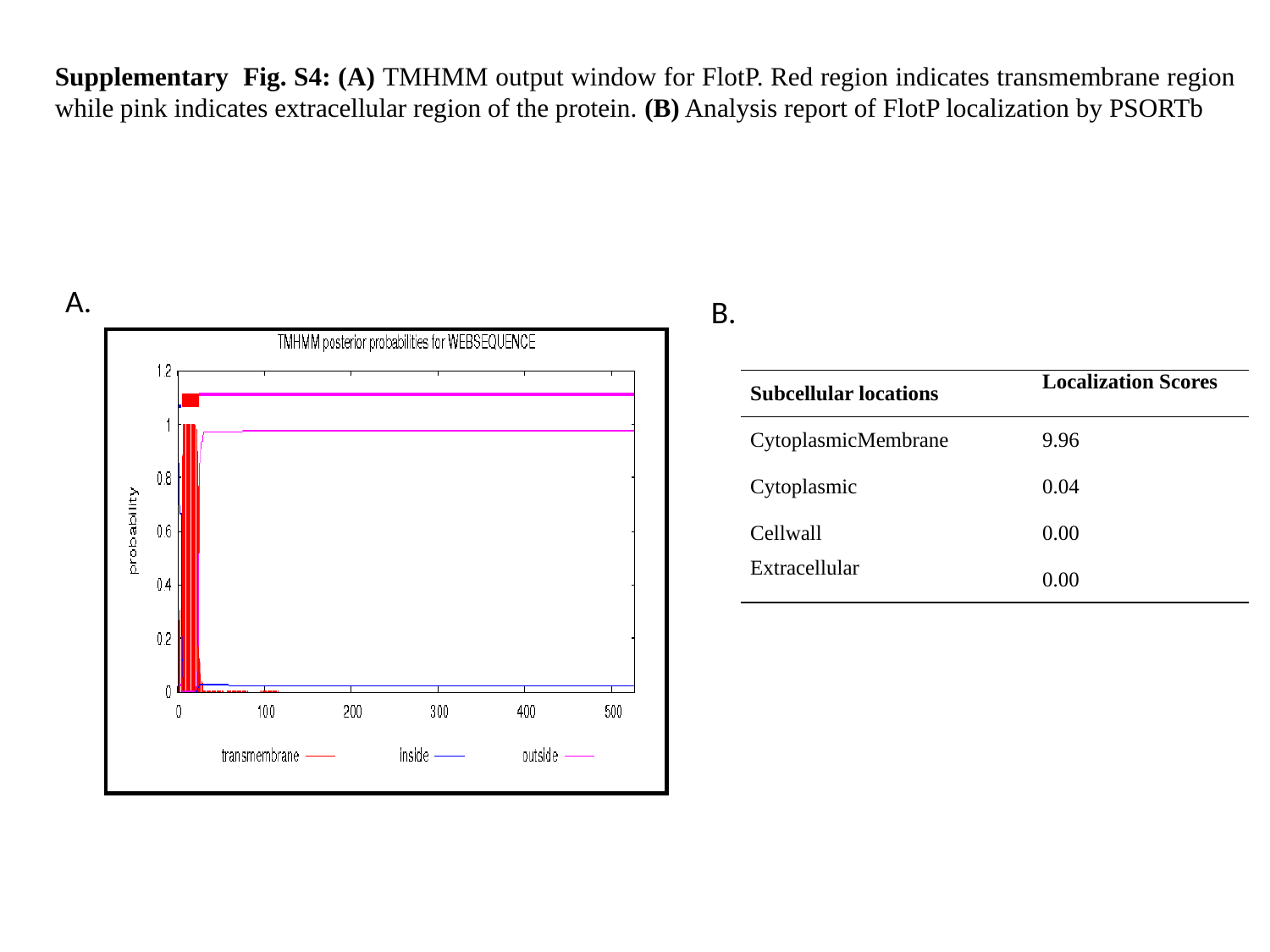

Supplementary Fig. S4: (A) TMHMM output window for FlotP. Red region indicates transmembrane region while pink indicates extracellular region of the protein. (B) Analysis report of FlotP localization by PSORTb
A.
B.
| Subcellular locations | Localization Scores |
| --- | --- |
| CytoplasmicMembrane | 9.96 |
| Cytoplasmic | 0.04 |
| Cellwall | 0.00 |
| Extracellular | 0.00 |
