# Supplementary material for: Identification of Novel Raft Marker Protein, FlotP in Bacillus anthracis
Source: Front Microbiol. 2016 Feb 17;7:169. doi: 10.3389/fmicb.2016.00169 (PMC4756111; doi:10.3389/fmicb.2016.00169)
Supplement: Supplementary file 5 [file Presentation3.PPT]

## Slide 1
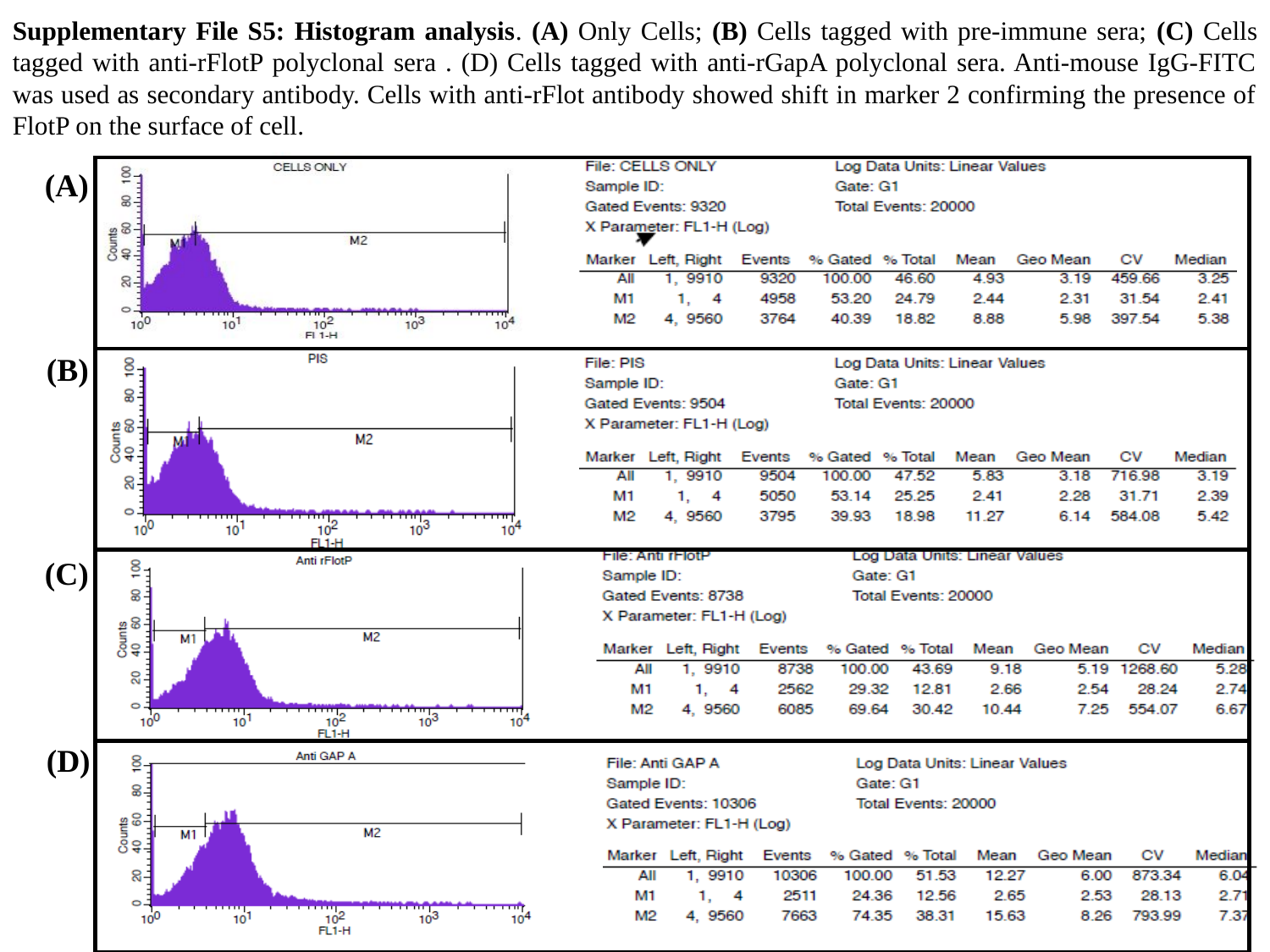

Supplementary File S5: Histogram analysis. (A) Only Cells; (B) Cells tagged with pre-immune sera; (C) Cells tagged with anti-rFlotP polyclonal sera . (D) Cells tagged with anti-rGapA polyclonal sera. Anti-mouse IgG-FITC was used as secondary antibody. Cells with anti-rFlot antibody showed shift in marker 2 confirming the presence of FlotP on the surface of cell.
(A)
(B)
(C)
(D)
