# Supplementary material for: Identification of Novel Raft Marker Protein, FlotP in Bacillus anthracis
Source: Front Microbiol. 2016 Feb 17;7:169. doi: 10.3389/fmicb.2016.00169 (PMC4756111; doi:10.3389/fmicb.2016.00169)
Supplement: Supplementary file 6 [file Presentation4.PPTX]

## Slide 1
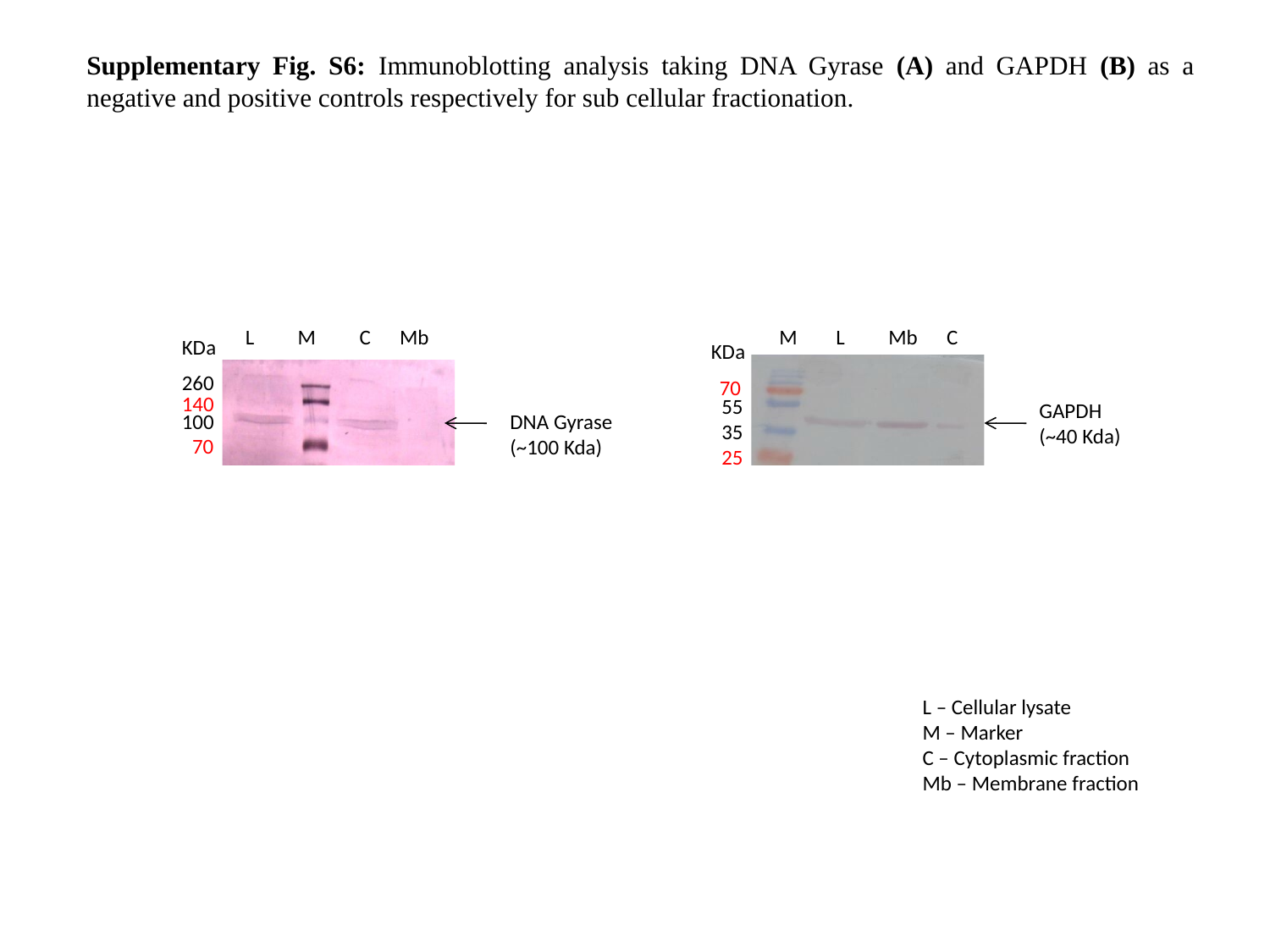

Supplementary Fig. S6: Immunoblotting analysis taking DNA Gyrase (A) and GAPDH (B) as a negative and positive controls respectively for sub cellular fractionation.
L M C Mb
KDa
260
140
100
70
 M L Mb C
KDa
55
25
35
70
GAPDH
(~40 Kda)
DNA Gyrase (~100 Kda)
L – Cellular lysate
M – Marker
C – Cytoplasmic fraction
Mb – Membrane fraction
