# Supplementary material for: Identification of Novel Raft Marker Protein, FlotP in Bacillus anthracis
Source: Front Microbiol. 2016 Feb 17;7:169. doi: 10.3389/fmicb.2016.00169 (PMC4756111; doi:10.3389/fmicb.2016.00169)
Supplement: Supplementary file 7 [file Presentation5.PPTX]

## Slide 1
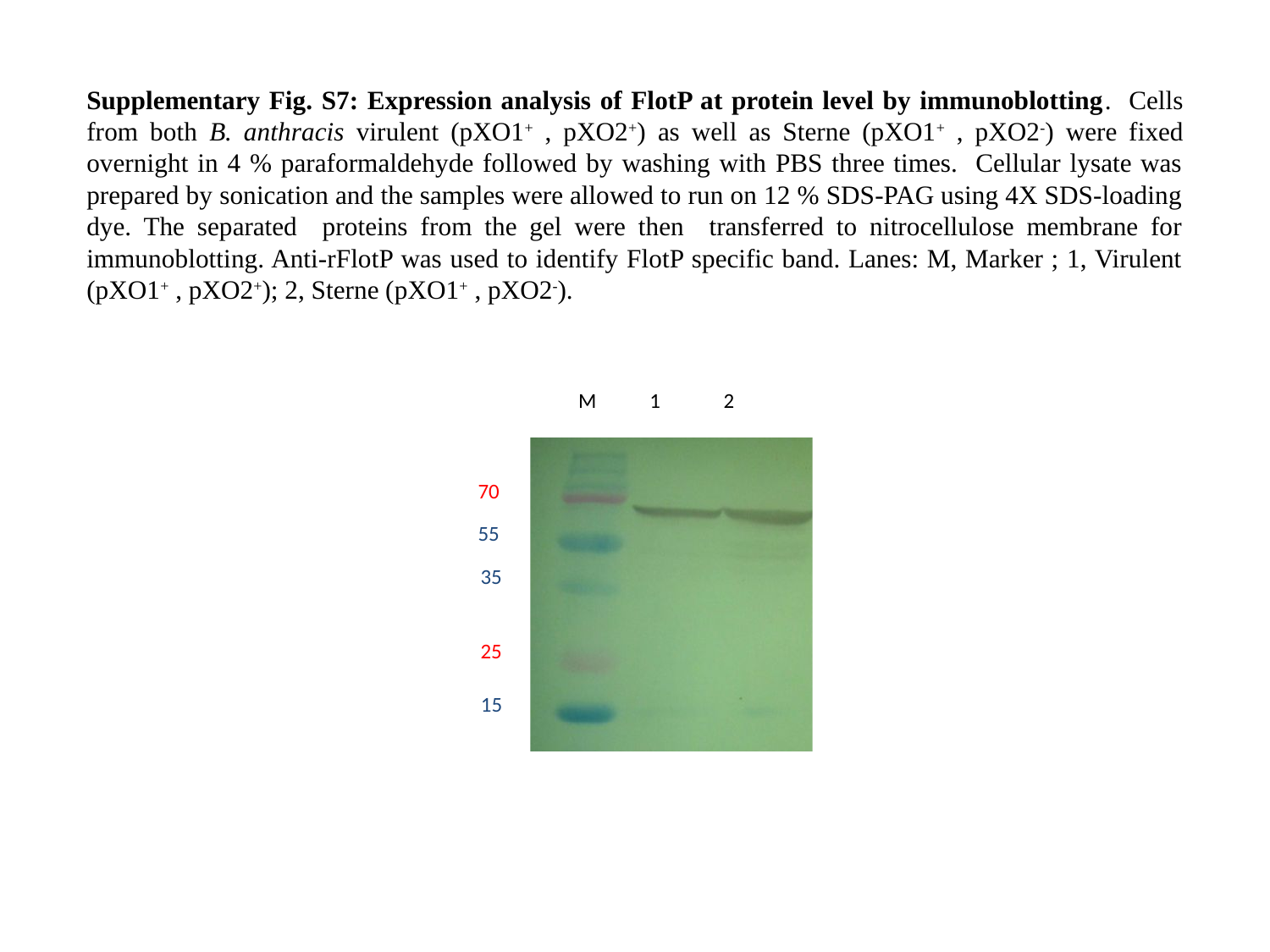

Supplementary Fig. S7: Expression analysis of FlotP at protein level by immunoblotting. Cells from both B. anthracis virulent (pXO1+ , pXO2+) as well as Sterne (pXO1+ , pXO2-) were fixed overnight in 4 % paraformaldehyde followed by washing with PBS three times. Cellular lysate was prepared by sonication and the samples were allowed to run on 12 % SDS-PAG using 4X SDS-loading dye. The separated proteins from the gel were then transferred to nitrocellulose membrane for immunoblotting. Anti-rFlotP was used to identify FlotP specific band. Lanes: M, Marker ; 1, Virulent (pXO1+ , pXO2+); 2, Sterne (pXO1+ , pXO2-).
 M 1 2
70
55
35
25
15

## Slide 2
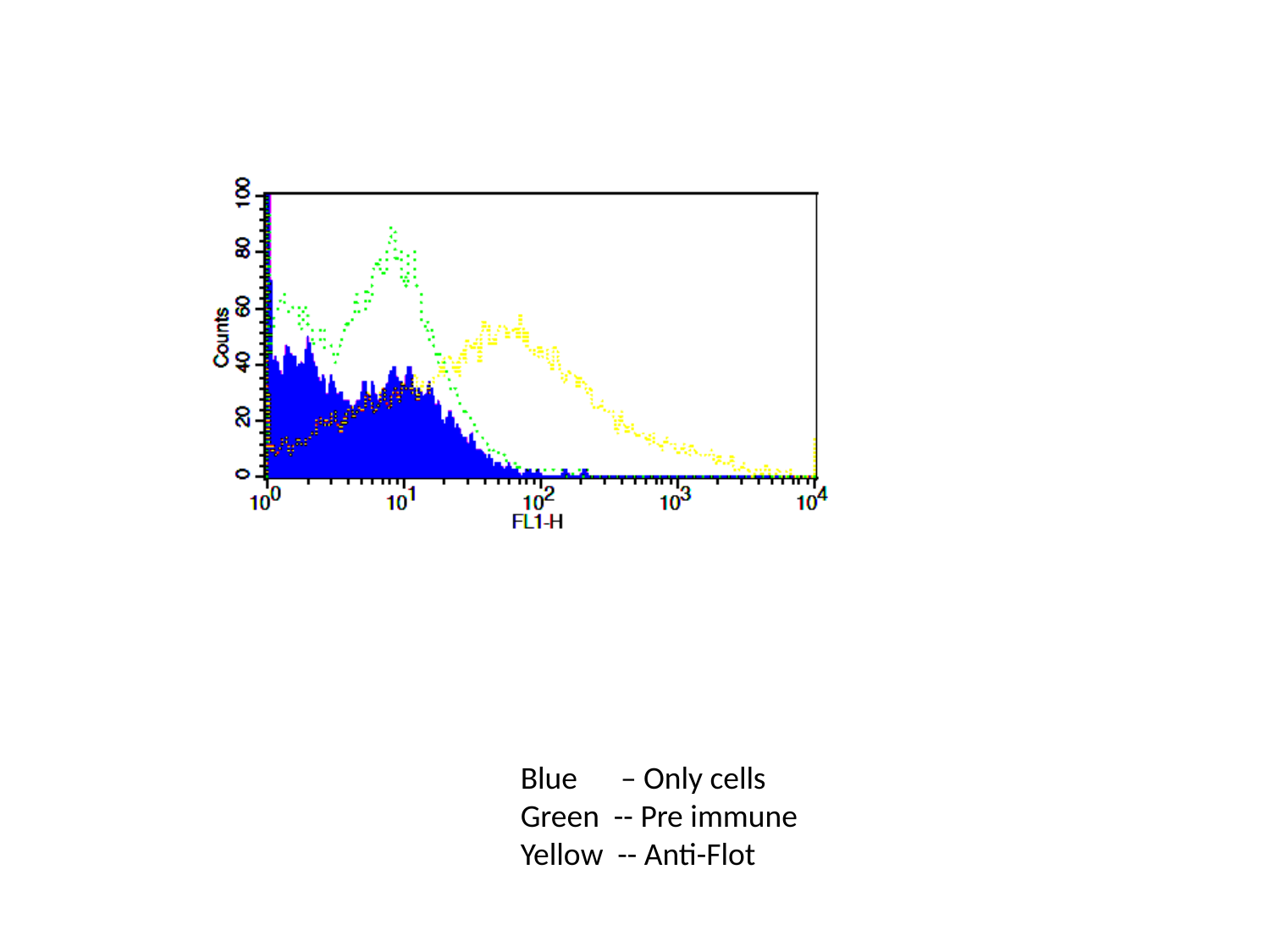

Blue – Only cells
Green -- Pre immune
Yellow -- Anti-Flot
